# Deciphering the Genetic Basis of Silkworm Cocoon Colors Provides New Insights into Biological Coloration and Phenotypic Diversification

**DOI:** 10.1093/molbev/msad017

**Published:** 2023-01-31

**Authors:** Yaru Lu, Jiangwen Luo, Erxia An, Bo Lu, Yinqiu Wei, Xiang Chen, Kunpeng Lu, Shubo Liang, Hai Hu, Minjin Han, Songzhen He, Jianghong Shen, Dongyang Guo, Nvping Bu, Ling Yang, Wenya Xu, Cheng Lu, Zhonghuai Xiang, Xiaoling Tong, Fangyin Dai

**Affiliations:** State Key Laboratory of Silkworm Genome Biology, Institute of Sericulture and Systems Biology, Southwest University, Chongqing, China; State Key Laboratory of Silkworm Genome Biology, Institute of Sericulture and Systems Biology, Southwest University, Chongqing, China; State Key Laboratory of Silkworm Genome Biology, Institute of Sericulture and Systems Biology, Southwest University, Chongqing, China; State Key Laboratory of Silkworm Genome Biology, Institute of Sericulture and Systems Biology, Southwest University, Chongqing, China; Key Laboratory of Sericulture Biology and Genetic Breeding, Ministry of Agriculture and Rural Affairs, College of Sericulture, Textile and Biomass Sciences, Southwest University, Chongqing, China; State Key Laboratory of Silkworm Genome Biology, Institute of Sericulture and Systems Biology, Southwest University, Chongqing, China; State Key Laboratory of Silkworm Genome Biology, Institute of Sericulture and Systems Biology, Southwest University, Chongqing, China; Key Laboratory of Sericulture Biology and Genetic Breeding, Ministry of Agriculture and Rural Affairs, College of Sericulture, Textile and Biomass Sciences, Southwest University, Chongqing, China; State Key Laboratory of Silkworm Genome Biology, Institute of Sericulture and Systems Biology, Southwest University, Chongqing, China; State Key Laboratory of Silkworm Genome Biology, Institute of Sericulture and Systems Biology, Southwest University, Chongqing, China; State Key Laboratory of Silkworm Genome Biology, Institute of Sericulture and Systems Biology, Southwest University, Chongqing, China; State Key Laboratory of Silkworm Genome Biology, Institute of Sericulture and Systems Biology, Southwest University, Chongqing, China; Key Laboratory of Sericulture Biology and Genetic Breeding, Ministry of Agriculture and Rural Affairs, College of Sericulture, Textile and Biomass Sciences, Southwest University, Chongqing, China; State Key Laboratory of Silkworm Genome Biology, Institute of Sericulture and Systems Biology, Southwest University, Chongqing, China; State Key Laboratory of Silkworm Genome Biology, Institute of Sericulture and Systems Biology, Southwest University, Chongqing, China; State Key Laboratory of Silkworm Genome Biology, Institute of Sericulture and Systems Biology, Southwest University, Chongqing, China; State Key Laboratory of Silkworm Genome Biology, Institute of Sericulture and Systems Biology, Southwest University, Chongqing, China; State Key Laboratory of Silkworm Genome Biology, Institute of Sericulture and Systems Biology, Southwest University, Chongqing, China; State Key Laboratory of Silkworm Genome Biology, Institute of Sericulture and Systems Biology, Southwest University, Chongqing, China; State Key Laboratory of Silkworm Genome Biology, Institute of Sericulture and Systems Biology, Southwest University, Chongqing, China; State Key Laboratory of Silkworm Genome Biology, Institute of Sericulture and Systems Biology, Southwest University, Chongqing, China; State Key Laboratory of Silkworm Genome Biology, Institute of Sericulture and Systems Biology, Southwest University, Chongqing, China; Key Laboratory of Sericulture Biology and Genetic Breeding, Ministry of Agriculture and Rural Affairs, College of Sericulture, Textile and Biomass Sciences, Southwest University, Chongqing, China; State Key Laboratory of Silkworm Genome Biology, Institute of Sericulture and Systems Biology, Southwest University, Chongqing, China; Key Laboratory of Sericulture Biology and Genetic Breeding, Ministry of Agriculture and Rural Affairs, College of Sericulture, Textile and Biomass Sciences, Southwest University, Chongqing, China

**Keywords:** Phenotypic variation, Domestication, Coloration, Flavonoids, Sugar transporter

## Abstract

The genetic basis of phenotypic variation is a long-standing concern of evolutionary biology. Coloration has proven to be a visual, easily quantifiable, and highly tractable system for genetic analysis and is an ever-evolving focus of biological research. Compared with the homogenized brown-yellow cocoons of wild silkworms, the cocoons of domestic silkworms are spectacularly diverse in color, such as white, green, and yellow-red; this provides an outstanding model for exploring the phenotypic diversification and biological coloration. Herein, the molecular mechanism underlying silkworm green cocoon formation was investigated, which was not fully understood. We demonstrated that five of the seven members of a sugar transporter gene cluster were specifically duplicated in the Bombycidae and evolved new spatial expression patterns predominantly expressed in silk glands, accompanying complementary temporal expression; they synergistically facilitate the uptake of flavonoids, thus determining the green cocoon. Subsequently, polymorphic cocoon coloring landscape involving multiple loci and the evolution of cocoon color from wild to domestic silkworms were analyzed based on the pan-genome sequencing data. It was found that cocoon coloration involved epistatic interaction between loci; all the identified cocoon color-related loci existed in wild silkworms; the genetic segregation, recombination, and variation of these loci shaped the multicolored cocoons of domestic silkworms. This study revealed a new mechanism for flavonoids-based biological coloration that highlights the crucial role of gene duplication followed by functional diversification in acquiring new genetic functions; furthermore, the results in this work provide insight into phenotypic innovation during domestication.

## Introduction

The rapid phenotypic variation in the short history of domestication is striking (Andersson and Georges 2004; Andersson and Purugganan 2022). Probably, due to artificial selection under the removal or relaxation of natural selection pressure, domestic animals tend to be more diverse in size, coloration, and other certain phenotypes, than wild animals, such as the dog's body shape and coat color, length, and curl (Cadieu et al. 2009; Bannasch et al. 2021)—the genetic basis for phenotypic diversification during domestication warrants further elucidation. Animal coloration, involving concealment, camouflage, courtship, signaling, and other critical biological processes, is an ongoing focus of biological research (Hubbard et al. 2010; Orteu and Jiggins 2020). Moreover, as a visible phenotypic marker, color plays a vital role in understanding genetic, developmental, and evolutionary theories (Orteu and Jiggins 2020). The cocoon color of *Bombyx mandarina*, commonly known as the wild silkworm, is homogenized brown-yellow. The cocoons of *Bombyx mori*, widely known as the domestic silkworm, are spectacularly diverse in color and can be divided into three categories according to the type and content of pigments: 1) yellow-red cocoons colored by carotenoids, such as golden, flesh, pink, rust-colored cocoons, etc., 2) green cocoons colored by flavonoids, the color from pale green to deep green, and 3) white cocoons with trace or no pigment (Xiang 2005; Sakudoh et al. 2007, 2010, 2013; Daimon et al. 2010). Uncovering the genetic basis for the formation and evolution of polymorphic cocoon color from wild to domestic silkworm provides an opportunity to further reveal the molecular basis underlying animal coloration and to understand the mechanism of phenotypic diversification.

In the silkworms producing colored cocoons, flavonoids and carotenoids from mulberry leaves are firstly absorbed by the midgut (MG) and then transported into the hemolymph (HC); next, the pigments are taken up by silk glands (SG) and bind to the silk protein; lastly, silk proteins bound with pigments are spun out by mature caterpillars to build colored cocoons. The chemical modification of pigments may accompany this process.

Proteins for intracellular and membrane transport of carotenoids are essential in forming yellow-red cocoons. The *Yellow blood* gene (*Y*, 2-28.6) encodes a carotenoid-binding protein (CBP) that is responsible for the intracellular transport of carotenoids in the MG and middle silk glands (MSG) (Sakudoh et al. 2007); this biological process can only occur in the absence of *Yellow inhibitor* (*I*, 9-16.2) with an inhibitory effect on *Y.* The *Yellow cocoon* gene (*C*, 12-7.2), encoding high-density lipoprotein receptor-2 (Cameo2), a membrane transporter belonging to the CD36 family, is involved in the membrane transport of lutein in MSG (Sakudoh et al. 2010). The *Flesh cocoon* gene (*F*, 6-34.7) encodes scavenger receptor class B member 15 (SCRB15), which also belongs to the CD36 family and differs from Cameo2 in that it has a higher affinity for β-carotene instead of lutein (Sakudoh et al. 2013). Classical genetic studies found additional loci contributing to yellow-red cocoons, such as the *Pink cocoon* (*Pk*, 2–?) and *Rusty cocoon* (*Rc*, 2–34.8) (Xiang 2005); inadequately, the molecular basis of these loci has not been revealed. Nevertheless, a preliminary panorama of yellow-red cocoon formation was established: in the absence of *I*, the *Y* with a broad spectrum of substrates for carotenoids is essential for transporting carotenoids from MG into the blood and thus underlies the formation of all yellow-red cocoons; in comparison, *C*, *F*, and possibly *Rc*, *Pk* are responsible for the selective absorption of carotenoids from the blood by MSG, which makes the cocoon exhibit different colors (Tsuchida and Sakudoh 2015).

More complex than the carotenoid coloration of cocoons, dietary flavonoids will undergo multiple chemical modifications in the silkworm tissues. *BmUGT10286*, the functional gene of a green cocoon-related locus named *Green b* (*Gb*, 7–7.0), encodes a glycosyltransferase that catalyzes the regioselective glycosylation of the quercetin 5-O position (Daimon et al. 2010). *BmP5CR1*, the gene responsible for the *Light green* cocoon (*Lg*, 6-?), encodes pyrroline-5-carboxylate reductase. Defects in *BmP5CR1* would lead to the accumulation of 1-pyrroline-5-carboxylic acid, thereby promoting the production of prolinylflavonols (Hirayama et al. 2018). BmUGT10286 and BmP5CR1 both catalyze the metabolic modification of flavonoids, thus enhancing the permeability of flavonoids in silkworms and ultimately contributing to the green cocoon. As important as they are, neither of them alone could lead to green cocoons; the assistance of proteins responsible for transporting flavonoids is necessary. However, the relevant genes remain unknown.

The above studies on colored cocoons were carried out on individual genes or loci in the domestic silkworm. Although it is generally accepted that color presentation is more complex and often involves the interactions of multiple loci (Dembeck et al. 2015; Orteu and Jiggins 2020), a systematic study on cocoon color involving multiple genes is lacking. Furthermore, along with the domestication from wild to domestic silkworm, the evolution process of cocoon color from homogenization to diversification is fascinating but largely unknown.

In this work, the molecular mechanism underlying green cocoon formation was further investigated, and membrane transporters involved in flavonoids absorption were first revealed in silkworms; it concerns a new mechanism for flavonoids-based biological coloration, which was previously largely unknown, especially in animals. Moreover, the polymorphic coloration landscape of silkworm cocoons involving multiple loci and the evolution of cocoon color from wild to domestic silkworms were analyzed based on the pan-genome sequencing data of domestic and wild silkworms, and a corresponding model was proposed, which provides new insight into our understanding of phenotypic variation during domestication.

## Results

### The Fifth Instar Is the Critical Period for the Silk Gland to Absorb Flavonoids

**Fig. 1.**
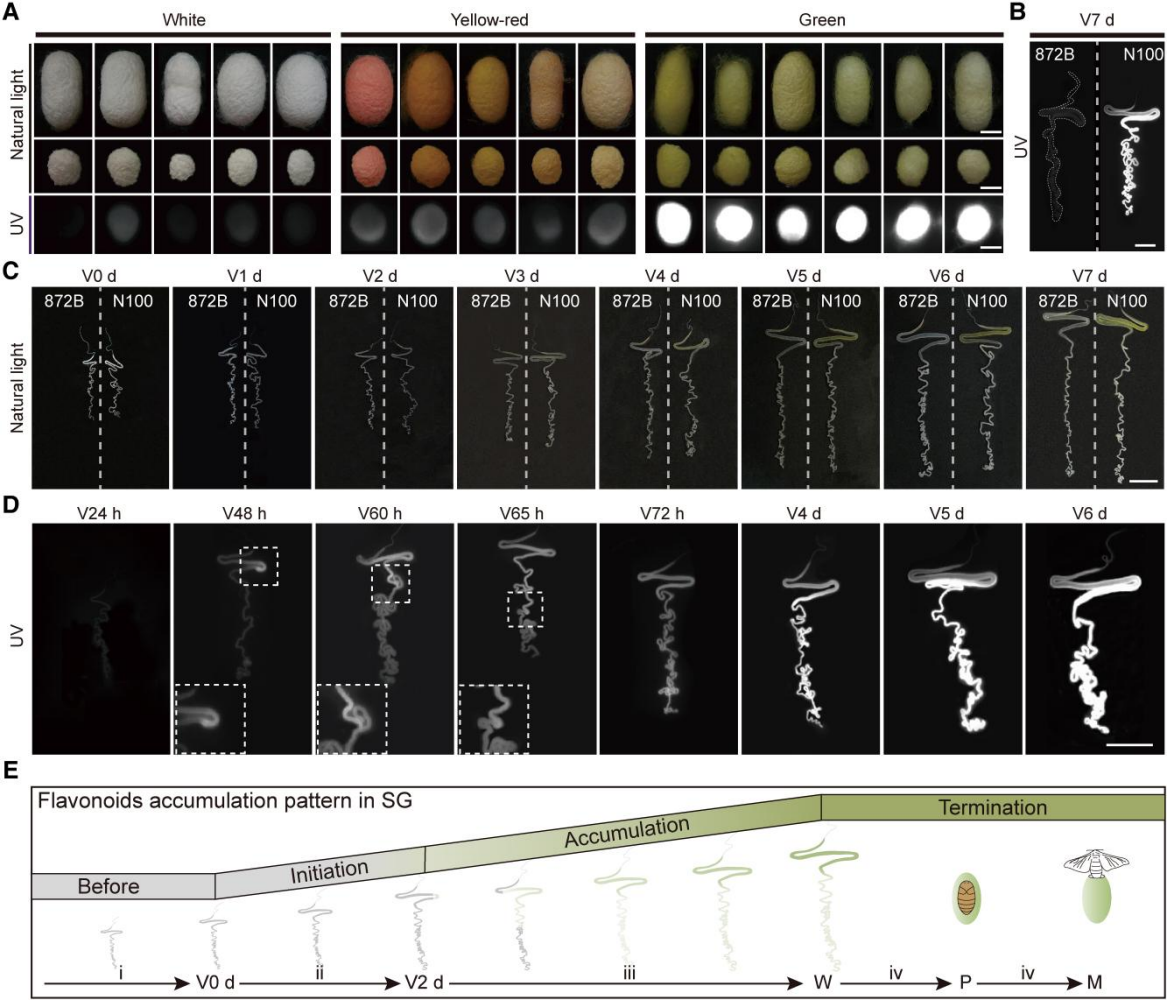
Phenotypic characteristics of colored cocoons and the accumulation process of flavonoids in SG. (*A*) White, yellow-red, and green cocoons of different strains under natural and UV light. Cocoons and cocoon fragments under natural light and cocoon fragments under UV are displayed from top to bottom. The exposure time under UV was 0.2 s. Scale bar, 1 cm. (*B*) SG of mature caterpillars of N100 and 872B under UV. The exposure time was 1.5 s. Scale bar, 2 cm. (*C*) SG of N100 and 872B at each developmental stage in the fifth instar under natural light. Scale bar, 2 cm. (*D*) SG of N100 at each developmental stage in the fifth instar under UV. Scale bar, 2 cm. The exposure time under UV was 1.5 s. V, fifth instar; d, day; h, hour. (*E*) Flavonoids accumulation pattern in SG. V, fifth instar; d, day; W, wandering; P, pupa; M, moth; i to iv, flavonoids accumulation stages in SG.

Flavonoids are the main pigment components in green cocoons of silkworms (Xiang 2005; Daimon et al. 2010; Hirayama et al. 2018); their accumulation pattern in silk glands (SG) remains unclear. Most flavonoids were found to fluoresce under UV (Havsteen 2002), as do some flavonoids in green cocoons (Daimon et al. 2010). Here, we explored whether it was feasible to determine the absorption and accumulation patterns of flavonoids in SG by observing the fluorescence. Among the three categories of cocoons (supplementary table S1, Supplementary Material online), the intense fluorescence under UV was specific to green cocoons colored by flavonoids (fig. 1*A*). Likewise, flavonoids-containing SG could also fluoresce under UV (fig. 1*B*); this permitted accurate observation of the accumulation process regarding flavonoids in SG.

The strains of N100 (green cocoon, *Gn*/*Gn*) and 872B (white cocoon, +*^Gn^*/+*^Gn^*) are a group of near-isogenic lines. In detail, N100 contains the *Gn* from the donor parent G200 (green cocoon, *Gn*/*Gn*) and has a genetic background of 872B. Therefore, except for different cocoon colors, N100 and 872B have similar developmental processes and were used for the following comparative study. Under natural light, the SG of N100 was observed to turn green on the third day of the fifth instar (final instar), and then the green color deepened day by day (fig. 1*C*). Next, this process was refined under UV. On the first day (24 h) of the fifth instar, no fluorescence signal of SG from N100 was observed. On the second day, the middle part of the MSG (synthesizing sericin) fluoresced. Over time, the fluorescence area expanded anteriorly and posteriorly. On the third day, the whole MSG and posterior silk glands (PSG, synthesizing fibroin) fluoresced, and the fluorescence intensity increased gradually, reaching a peak on the seventh day of the fifth instar (fig. 1*B*and*D*), the moment that the mature caterpillars begin to spin silk and build cocoons, known as the wandering stage. These data suggested that the fifth instar is the critical period for SG to take up flavonoids: in the fifth instar, the flavonoids in SG start to be present and then gradually increase. According to these results, we divided the accumulation process of flavonoids in SG into four stages: stage 1, “Before”, before the fifth instar; stage 2, “Initiation”, the first 2 days of the fifth instar; stage 3, “Accumulation”, from the third day of the fifth instar to wandering stage; stage 4, “Termination”, the wandering stage and the whole development period after that (fig. 1*E*).

### Genome-Wide Association Analysis of Green Cocoons and Positional Cloning of the *Gn* Locus

**Fig. 2.**
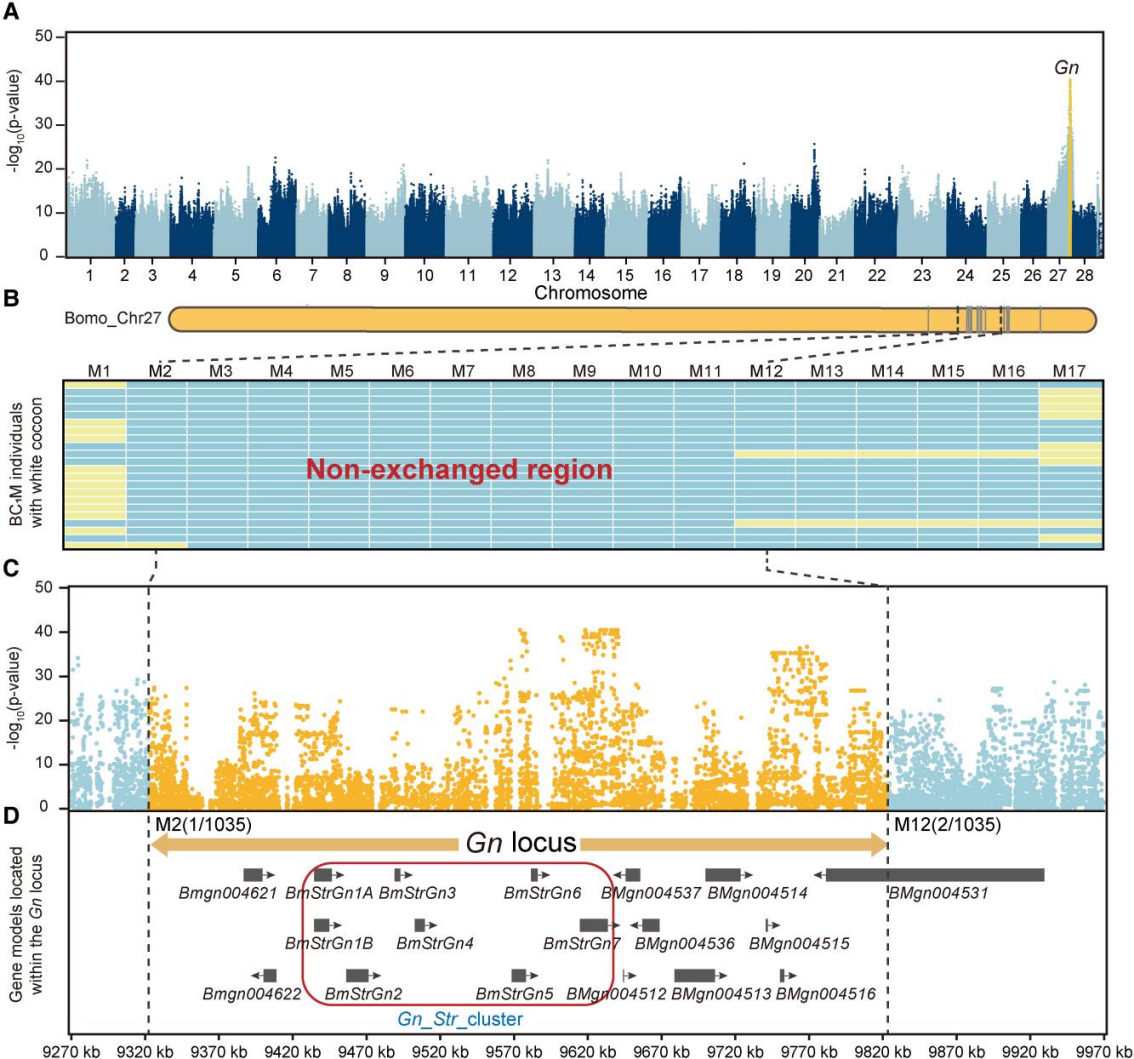
GWAS of the green cocoon and positional cloning of the *Gn*. (*A*) Manhattan plot of GWAS. (*B*) Positional cloning of *Gn.* The vertical lines indicate 17 polymorphic molecular markers on Bomo_Chr27. The genotypes of each polymorphic molecular marker in partial individuals of BC_1_*M* are displayed. The yellow and light blue squares represent heterozygous and *+^Gn^* homozygous genotypes. (*C*) and (*D*) Regional Manhattan plot and gene models around the *Gn* locus. The region separated by broken black lines indicates the *Gn* locus narrowed by positional cloning; orange dots in (*A*) and (*C*) represent the SNPs in this region. The dark gray rectangular blocks in (*D*) indicate the gene models. The arrows show the direction of gene transcription. A rounded rectangle with a red border encircles the *Gn_Str*_cluster.

To identify the loci responsible for green cocoons, we performed a genome-wide association analysis (GWAS) using the single nucleotide polymorphisms (SNPs, a total of 12,0 27,355) of 60 and 51 silkworm strains with white and green cocoons, respectively (supplementary table S2, Supplementary Material online). Before this work, we identified a green cocoon-related locus on chromosome 27 through classical genetic research and named it *New Green Cocoon* (*Gn*) (Dai 2008). Here, the Manhattan plot of the GWAS indicated that a single region on chromosome 27 exhibited the most significant association signal (fig. 2*A*); thus, it was identified as the candidate region for *Gn.* Then, fine mapping based on linkage analysis was performed to check the range of candidate genes of *Gn*. The male F_1_ heterozygous of G200 and 872B were backcrossed with 872B to obtain the population (BC_1_M) for positional cloning of *Gn* (supplementary fig. S1, Supplementary Material online). Genomic DNA of 1,035 BC_1_M progenies with white cocoons was used for the linkage analyses between *Gn* and 17 polymorphic molecular markers (supplementary table S3, Supplementary Material online) on chromosome 27 (fig. 2*B*). Finally, the *Gn* was narrowed to a 500-kb genomic region that completely overlapped with the most significant region obtained by GWAS (fig. 2*A*and*C*). There were 17 protein-coding genes in this region, including a sugar transporter (*Str*) gene cluster whose members belong to the major facilitator superfamily (MFS; fig. 2*D*, supplementary table S4, Supplementary Material online); this *Str* gene cluster was abbreviated as *Gn_Str_*cluster henceforward. The *Gn_Str*_cluster harbored seven members named *BmStrGn1* to *BmStrGn7*, in which *BmStrGn1* had two transcripts, *BmStrGn1A* and *BmStrGn1B* (fig. 2*D*).

### Five Members of *Gn_Str*_Cluster Were Strong Candidate Genes for *Gn*

**Fig. 3.**
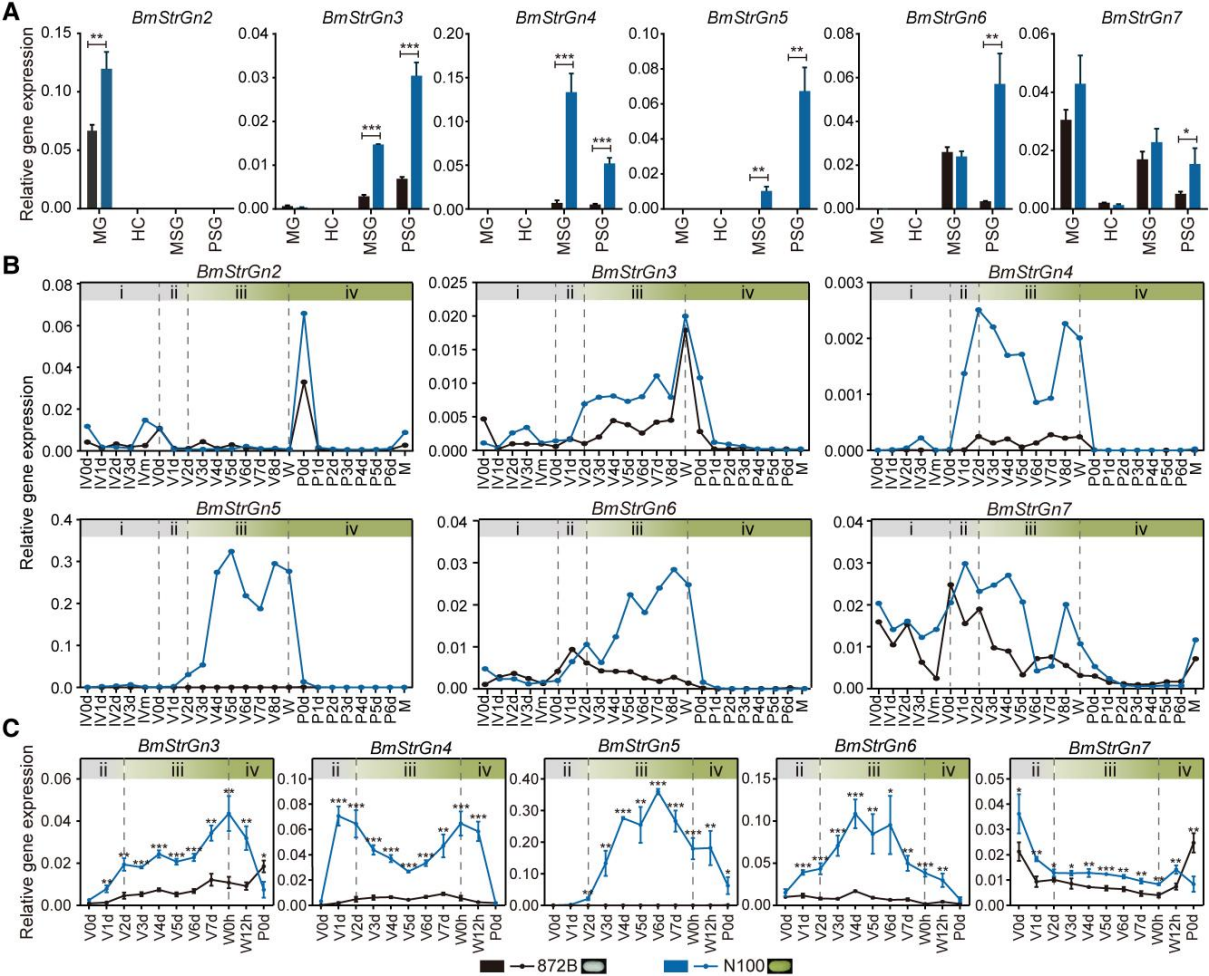
Spatiotemporal expression of *Strs* within the *Gn* locus. (*A*) Expression of *Strs* in the MG, HC, and SG of N100 and 872B caterpillars on the third day of the fifth instar. (*n* = 3 biological replicates). (*B*) and (*C*) Temporal expression of *Strs* in the entire silkworm body and SG of N100 and 872B, respectively. IV, fourth instar; V, fifth instar; W, wandering; P, pupa; M, moth; m, molting; d, day; h, hour; i to iv, flavonoids accumulation stages in SG. *, **, and *** indicate that the *P* values of unpaired, two-tailed *t*-tests are less than 0.05, 0.01, and 0.001, respectively. Error bars represent mean ± SD (*n* = 3 biological replicates).

The expression patterns of genes typically correspond to their biological functions. Hence, the genes within *Gn* with spatiotemporal expression patterns that fit the accumulation process of flavonoids in SG were screened. Still, considering that N100 and 872B have similar genetic backgrounds as a set of near-isogenic lines for the *Gn*, the comparative experiments for the expression investigation were carried out between them. Firstly, the expression of non-*Str* genes was absent in any of the MG, HC, and SG, the critical tissues for flavonoids absorption and metabolism, or not significantly different between N100 and 872B (supplementary fig. S2, Supplementary Material online). So, we focused on the *Gn_Str*_cluster located in a genomic region with a high degree of linkage disequilibrium (supplementary fig. S3, Supplementary Material online). Except for the *BmStrGn1*, *BmStrGn2* to *BmStrGn7* were highly expressed in MG or SG, and their expression level in SG of N100 was significantly higher than that in 872B (fig. 3*A*). Additionally, except for the predominant expression of *BmStrGn2* in the new pupae that had just metamorphosed from caterpillars, *BmStrGn3* to *BmStrGn7* were highly or specifically expressed during the fifth instar (the critical period for SG to take up flavonoids), both in the entire silkworm body (fig. 3*B*) and in the SG (fig. 3*C*) of N100. In the 872B, they were low or barely expressed (fig. 3*B*and*C*). In brief, the expression patterns of *BmStrGn3*, *BmStrGn4*, *BmStrGn5*, *BmStrGn6*, and *BmStrGn7* (collectively called *Gn_Strs*) highly fitted with the accumulation patterns of flavonoids in SG.

**Fig. 4.**
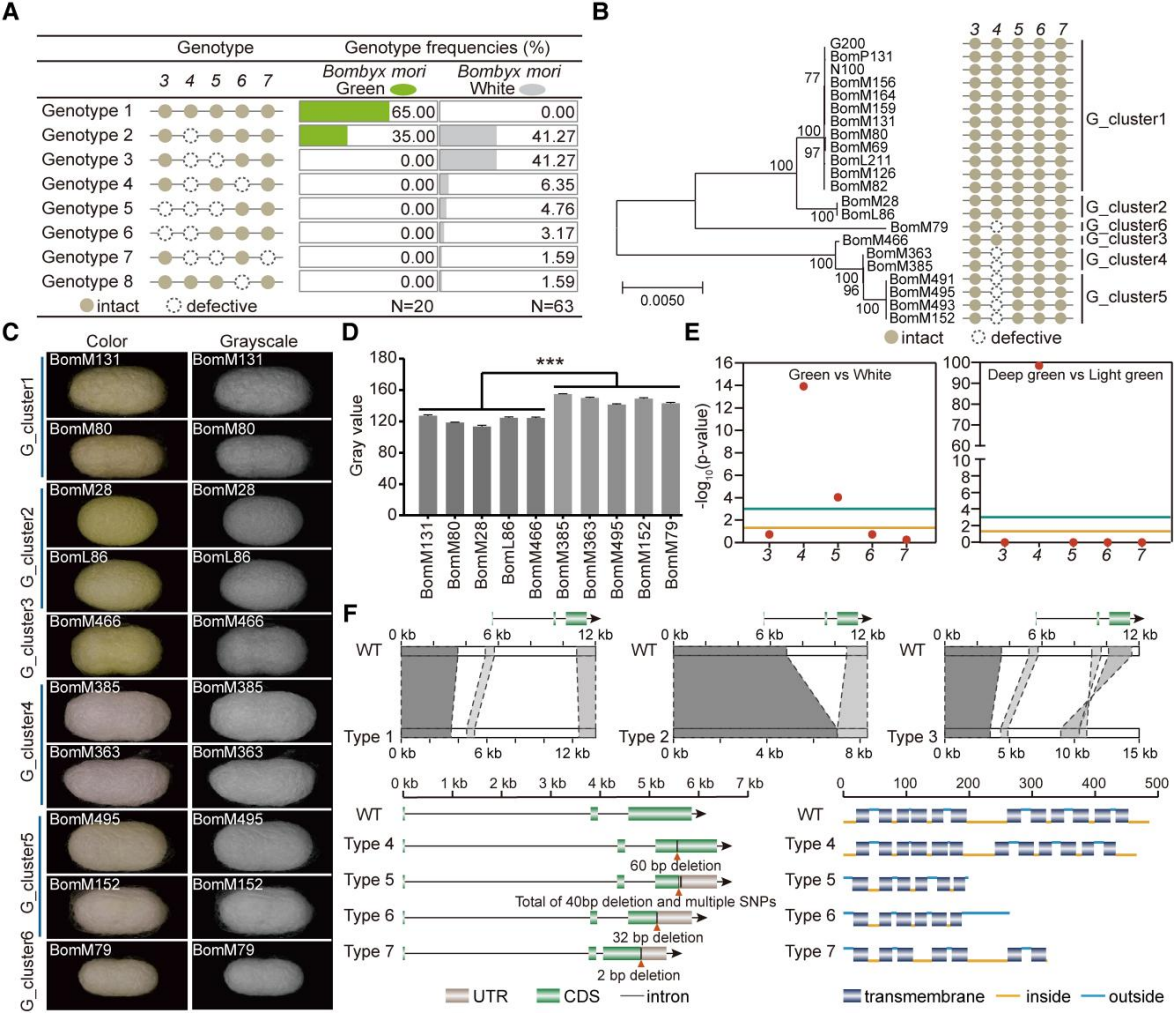
Association of the coding potential of each *Gn_Str* with green cocoons. (*A*) Coding potential of *Gn_Strs* in multiple domestic silkworm strains. The prefix “*BmStrGn*” of the gene ID is omitted. (*B*) Phylogenetic tree for the nucleotide sequences in the *Gn_Strs* genomic regions of strains with green cocoons. The percentages of replicate trees in the bootstrap test (1,000 replicates) are shown next to the branches. (*C*) Color and grayscale photos of the representative green cocoons for each cluster of *Gn_Strs*. Scale bar, 1 cm. (*D*) Gray values of green cocoons. Error bars represent mean ± SD (*n* = 3 technical replicates). *** indicate that the *P*-values of unpaired, two-tailed t-tests are less than 0.001. (*E*) Association of the coding potential of each *Gn_Str* with green cocoon and the shades of the green cocoon. In the one-way ANOVA, the genotype of “intact” and “defective” were assigned a value of 1 and 0, respectively. If the *P*-value of one-way ANOVA was 0, then -log_10_ (*P*-value) was recorded as 100. (*F*) Multiple types of sequence variation lead to functional defects of *BmStrGn4* in the pale green and white cocoon populations. In Type 1, Type 2, and Type 3, large segments of sequence substitutions, deletions, and inversions occurred in the regions from the first intron to the third exon of *BmStrGn4*, respectively; in Type 4 to 7, several nucleotides or small fragment sequences were deleted in the third exon of *BmStrGn4*; all these variations result in *BmStrGn4* being incapable of encoding proteins with complete functional domains.

Further, the *Gn_Strs* were genotyped in multiple domestic silkworm strains to investigate the cocoon color differentiation accompanying their genotype change in *Gn_Strs*. The putative regulatory regions (defined as introns, upstream and downstream 5-kb genomic regions of each gene) and coding regions of each *Gn_Str* were of interest. First, there were SNPs highly associated with green cocoons in the coding and regulatory regions of each *Gn_Str* (supplementary fig. S4, Supplementary Material online). The variants located in the regulatory region may be responsible for the differential expression of *Gn_Strs* between strains building white and green cocoons. We next investigated whether variants in the coding regions resulted in changes in the functional domains of Gn_Strs. A total of 83 domestic silkworm strains were surveyed for the coding regions and the corresponding functional domains of Gn_Strs, including 63 and 20 strains with white and varying degrees of green cocoons, respectively. Among the 20 investigated domestic silkworm strains spinning a green cocoon, 13 samples had intact *Gn_Strs* (genotype1), that is each *Gn_Str* had the potential to encode a protein with a complete MFS domain (fig. 4*A*, supplementary table S6, Supplementary Material online). The other seven strains were deficient in *BmStrGn4* (genotype2; fig. 4*A*, supplementary table S6, Supplementary Material online). Phylogenetic analysis for the nucleotide sequences of genomic regions in their *Gn_Strs* showed that these 20 silkworm strains could be clustered into six groups, that is G_cluster1 to G_cluster3 with genotype1 and G_cluster4 to G_cluster6 with genotype2, respectively (fig. 4*B*). There was a significant difference between cocoons with “genotype 1” and “genotype 2” in terms of green depth (fig. 4*C*and*D*). In all 63 investigated strains with white cocoons, one or more members of *Gn_Strs* were defective (fig. 4*A*, supplementary table S6, Supplementary Material online). Next, the association between the coding potential of each *Gn_Str* and green cocoon was analyzed. Remarkably, *BmStrGn4* exhibited the highest correlation with both the green cocoon phenotype and the shade of green cocoon (fig. 4*E*); it was defective in almost all silkworm strains with a white or pale green cocoon due to multiple complex sequence variations, including substitutions, deletions, and inversions (fig. 4*F*). These results suggested that *Gn_Strs*, especially *BmStrGn4*, were strong candidate genes for *Gn*.

### Five *Gn_Strs* Contribute to the Green Cocoon with Different Efficacy

**Fig. 5.**
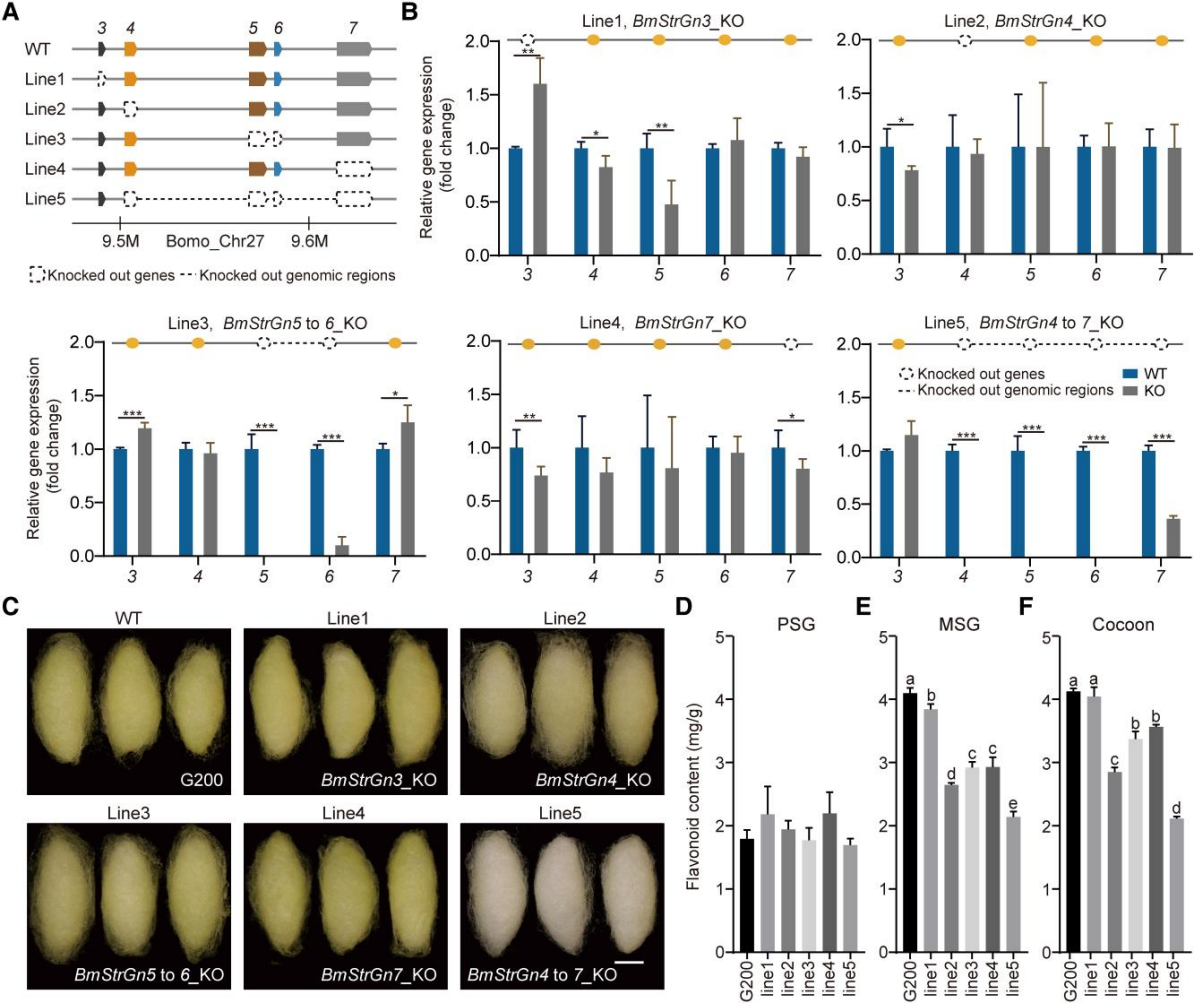
CRISPR/Cas9 mediated single or multiple *Gn_Strs* knockouts. (*A*) Schematic diagram of *Gn_Strs* knockout. The prefix “*BmStrGn*” of the gene ID is omitted. WT, wild type, strain G200. (*B*) Expression fold change of *Gn_Strs* in each *Gn_Str* knockout line. Error bars represent mean ± SD (*n* = 4 to 6 biological replicates). *, **, and *** indicate that the *P* values of unpaired, two-tailed *t*-tests are less than 0.05, 0.01, and 0.001, respectively. (*C*) Cocoons of wild-type and knockout lines. KO, knockout; scale bar, 1 cm. (*D*–*F*) Total flavonoids content in PSG, MSG, and cocoons of each knockout line. PSG and MSG were dissected from the mature silkworms of each knockout line and G200. Data are presented as mean ± SD (*n* = 3 biological replicates); significant differences (*P* values of unpaired, two-tailed *t*-tests are less than 0.05) between the two data sets are marked with different letters.

To verify our hypothesis that *Gn_Strs*, as strong candidate genes for *Gn*, play an essential role in the uptake of flavonoids in SG, single and multiple *Gn_Strs* were knocked out in strain G200 using CRISPR/Cas9-mediated gene editing (fig. 5*A*, supplementary fig. S5, Supplementary Material online). According to the spatiotemporal expression pattern of *Gn_Strs*, the following strategies were formulated for the functional research of *Gn_Strs*: the expression level of *BmStrGn3* peaked during the wandering stage (fig. 3*B*and*C*), during which the accumulation of flavonoids in SG also reached its peak, and it was knocked out alone as line 1. *BmStrGn4* was highly expressed in the SG throughout the entire fifth instar (fig. 3*C*), and it was knocked out alone as line 2. *BmstrGn5* and *BmstrGn6* were highly expressed in stage 3 (Accumulation) (fig. 3*B*and*C*), when the SG of caterpillars grows rapidly, and the accumulation of flavonoids continues to increase. Thus, these two genes were knocked out together as line 3. Next, *BmStrGn7*, the only member of *Gn_Strs* expressed in the MG (fig. 3*A*), the tissue where flavonoids are first absorbed and metabolized, was knocked out alone as line 4. Finally, to further explore the function of the *Gn_Strs*, the 122-kb genomic region harboring *BmStrGn4*, *BmStrGn5*, *BmStrGn6*, and *BmStrGn7* was knocked out as line 5.

We first investigated whether there was transcriptional compensation among *Gn_Strs*, which was detected between highly similar duplicate copies (Xia et al. 2021), known as transcriptional adaptation (Jakutis and Stainier 2021). After *Gn_Strs* were knocked out in each line, the unmanipulated members had no evident upregulation to compensate (fig. 5*B*); this implied that each knockout line’s phenotype corresponds to the manipulated gene's function. As expected, the knockout of *Gn_Strs* led to the fading of cocoon color and the decrease of total flavonoids content in SG and cocoons to different degrees. After *BmStrGn4* was knocked out individually (line 2), the cocoon color was lighter than those of other *Gn_Strs* and G200 (fig. 5*C*). Notably, the cocoon color of line 5, with the large fragment knockout of the *Gn_Strs* genomic region, was pale green and close to white (fig. 5*C*). There was no significant change in the content of total flavonoids in the PSG of mature silkworms of each knockout line compared with G200 (fig. 5*D*); the reasons for this need to be investigated in future work, the continuous migration of flavonoids in PSG along with silk fibroin to MSG during the fifth instar may be one of the triggers (Tashiro and Otsuki 1970). In MSG and cocoons, the total flavonoids content from high to low was in line 1, line 3 or 4, line 2, and line 5 (fig. 5*E*and*F*). These results indicated that *Gn_Strs* work together but with different efficiency to facilitate the uptake of flavonoids in SG, in which *BmStrGn4* was a major gene. The results echoed that *BmStrGn4* exhibited the highest correlation with the green cocoons (fig. 4*E*).

### *Gn_Strs* Are Bombycidae-Specific and Undergo Sub/Neofunctionalization

Strs are well known for their critical role in transporting nutrients, such as glucose (Chen et al. 2015). Our results demonstrated that five of the seven members of *Gn_Str_*cluster, that is *Gn_Strs*, have similar functions in facilitating the absorption of flavonoids and thus contributing to the green cocoon. Therefore, we carried out the following studies to clarify the origination and functional evolution of *Gn_Str*_cluster.

**Fig. 6.**
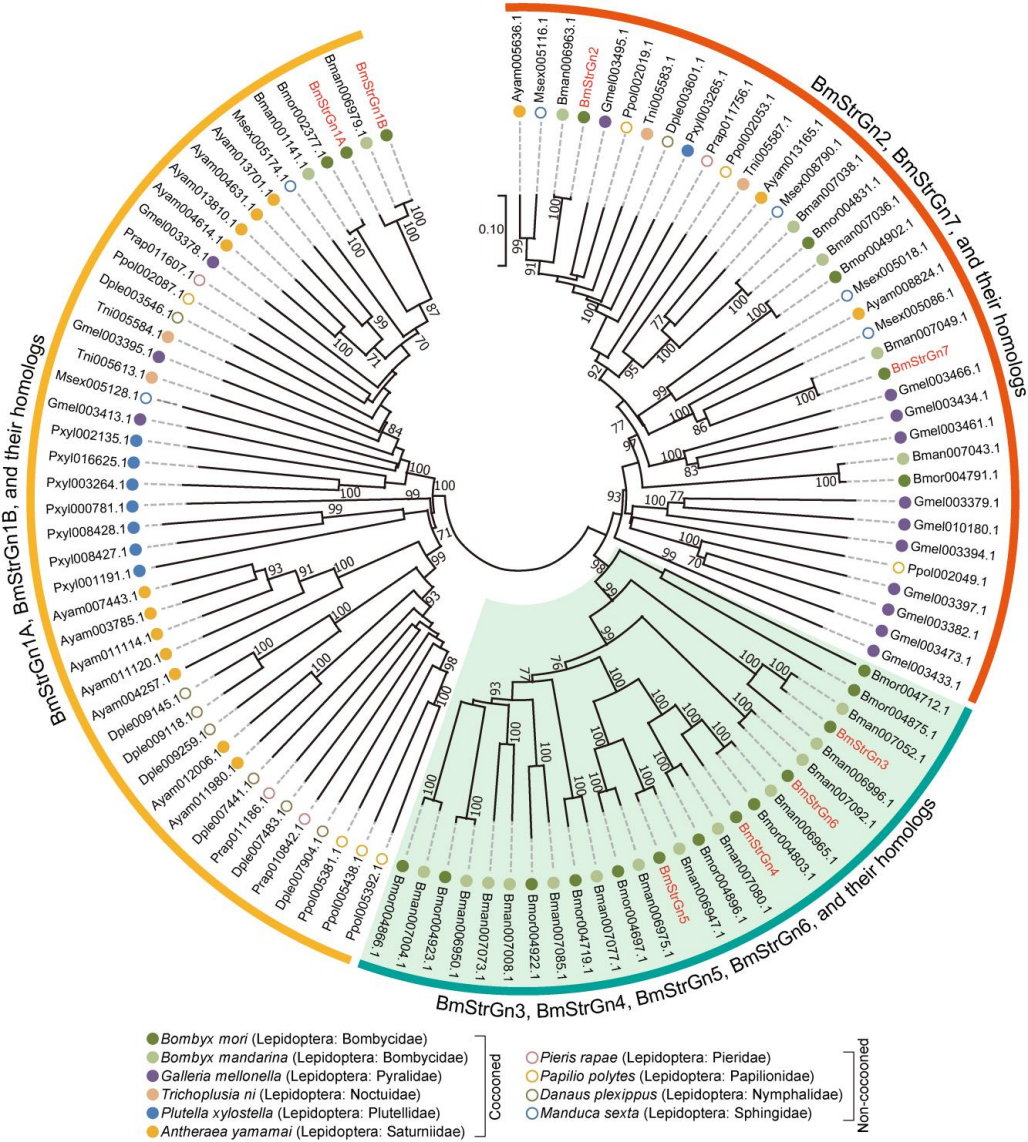
Phylogenetic tree of Gn_Str_cluster members in *B. mori* and their homologs in five other cocooned and four noncocooned Lepidoptera insects. All Strs encoded by these Lepidoptera's genome were identified and carried out the phylogenetic analysis. Only the subtrees in which Gn_Str_cluster members are located are displayed. More detailed information is available in supplementary figure S7, Supplementary Material online.

**Fig. 7.**
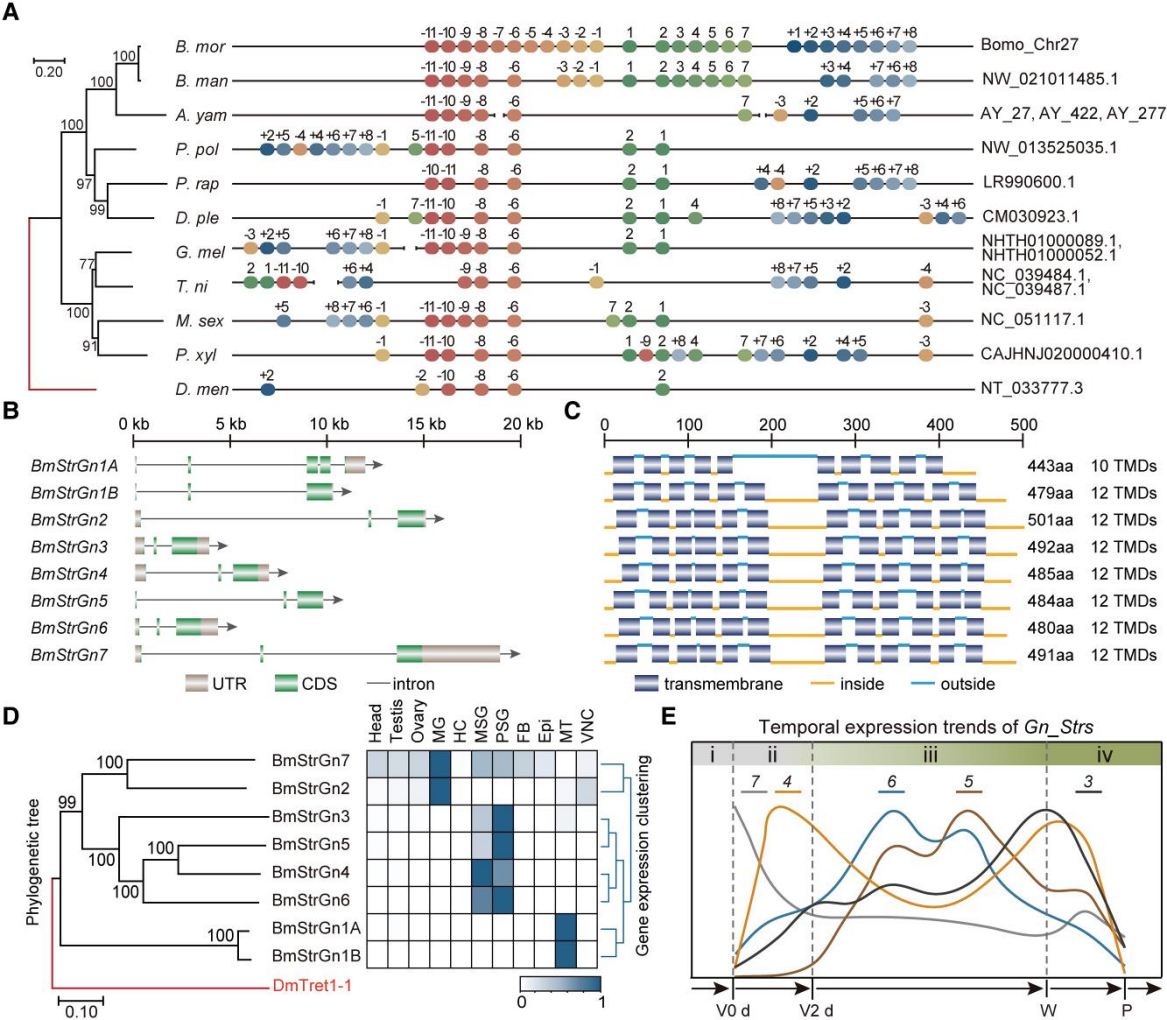
Evolution of *Gn_Str_*cluster. (*A*) Syntenic analysis of *Gn_Str_*cluster in Lepidoptera. Only genes homologous to genes around the *Gn* locus are shown for each species. Information on the location of each gene in each species is provided in supplementary tables S8 and S9, Supplementary Material online. *Gn_Str_*cluster members and their homologs are labeled 1–7; genes upstream of the *Gn_Str*_cluster and their homologs are labeled −11 to −1; genes downstream of the *Gn_Str*_cluster and their homologs are labeled +1 to +8. The phylogenetic tree was constructed using the sequences of the coding regions of *Strs* within the *Gn* locus. In the tree building, the sequence of the homologous region in *Drosophila melanogaster* (*D. men*) was used as an outgroup. The percentages of replicate trees in the bootstrap test (1,000 replicates) are shown next to the branches. *B. mor*, *Bombyx mori*; *B. man*, *Bombyx mandarina*; *P. rap*, *Pieris rapae*; *P. xyl*, *Plutella xylostella*; *D. ple*, *Danaus plexippus*; *M. sex*, *Manduca sexta*; *A. yam*, *Antheraea yamamai*; *G. mel*, *Galleria mellonella*; *P. pol*, *Papilio polytes*; *T. ni*, *Trichoplusia ni*. (*B*) Gene structure of *Gn_Str_*cluster members. (*C*) Transmembrane domains of Strs corresponding to (*B*). (*D*) Phylogenetic tree and spatial expression heat map of *Strs*. The phylogenetic tree of Strs was constructed using amino acid sequences. Dotted lines assign branches of BmStrGn1*A* and BmStrGn1*B* encoded by one gene. The percentages of replicate trees in the bootstrap test (1,000 replicates) are shown next to the branches. The protein sequence of facilitated trehalose transporter Tret1-1 in *Drosophila melanogaster*, DmTret1-1, was used as an outgroup in the tree building. Total RNAs were extracted from the tissues of G200 caterpillars on the third day of the fifth instar. FB, fat body; Epi, epidermis; MT, malpighian tubules; VNC, ventral nerve cord. (*E*) Complementary temporal expression of *Gn_Strs.* The Gene expression trends were graphed based on figure 3*C*. The prefix “*BmStrGn*” of the gene ID is omitted. V, fifth instar; W, wandering; P, pupa; d, day; i to iv, flavonoids accumulation stages in SG.

Firstly, all Strs encoded by the genome of cocooned and noncocooned Lepidoptera belonging to different families were investigated. All the species surveyed contained more than 150 *Str* duplicates (supplementary fig. S6, Supplementary Material online). Most Strs, including BmStrGn1 and BmStrGn2, were conserved among species (fig. 6, supplementary fig. S7, Supplementary Material online); they probably emerged before the divergence of these species. Strikingly, four Gn_Strs, that is BmStrGn3, BmStrGn4, BmStrGn5, and BmStrGn6, are specific to the Bombycidae (fig. 6); it was also supported by the syntenic analysis of *Gn_Str_*cluster and its upstream and downstream genes in Lepidoptera (fig. 7*A*). These results implied the specific function of Gn_Strs in Bombycidae.

All the members of the *Gn_Str*_cluster except for *BmStrGn1A* shared a similar gene structure with three exons, and the lengths of corresponding exons were similar and contained 12 canonical transmembrane domains of MFS (fig. 7*B*and*C*). With the gene duplication and the accumulation of divergent mutations, the expression profiles of these *Strs* were differentiated. Moreover, *Strs* with high sequence similarity had similar spatial expression patterns: BmStrGn1A and BmStrGn1B, with 91% identity, shared the same regulatory region and were specifically expressed in malpighian tubules. BmStrGn2 and BmStrGn7, with 56% identity, were highly expressed in the MG. BmStrGn3, BmStrGn4, BmStrGn5, and BmStrGn6, with 47% to 69% identity, were specifically expressed in SG (fig. 7*D*, supplementary figs. S8 and S9, Supplementary Material online). Moreover, *Gn_Strs* showed complementary temporal expression patterns in SG: exhibiting “as one falls, another rises,” with their expression levels peaked at different flavonoids accumulation stages in SG of the fifth instar caterpillars (fig. 7*E*). These results indicated the fate of *Gn_Strs* to undergo neofunctionalization accompanied by subfunctionalization.

### Polymorphic Cocoon Coloring Landscape Involving Multiple Loci and Evolution of Cocoon Color from Wild to Domestic Silkworm

**Fig. 8.**
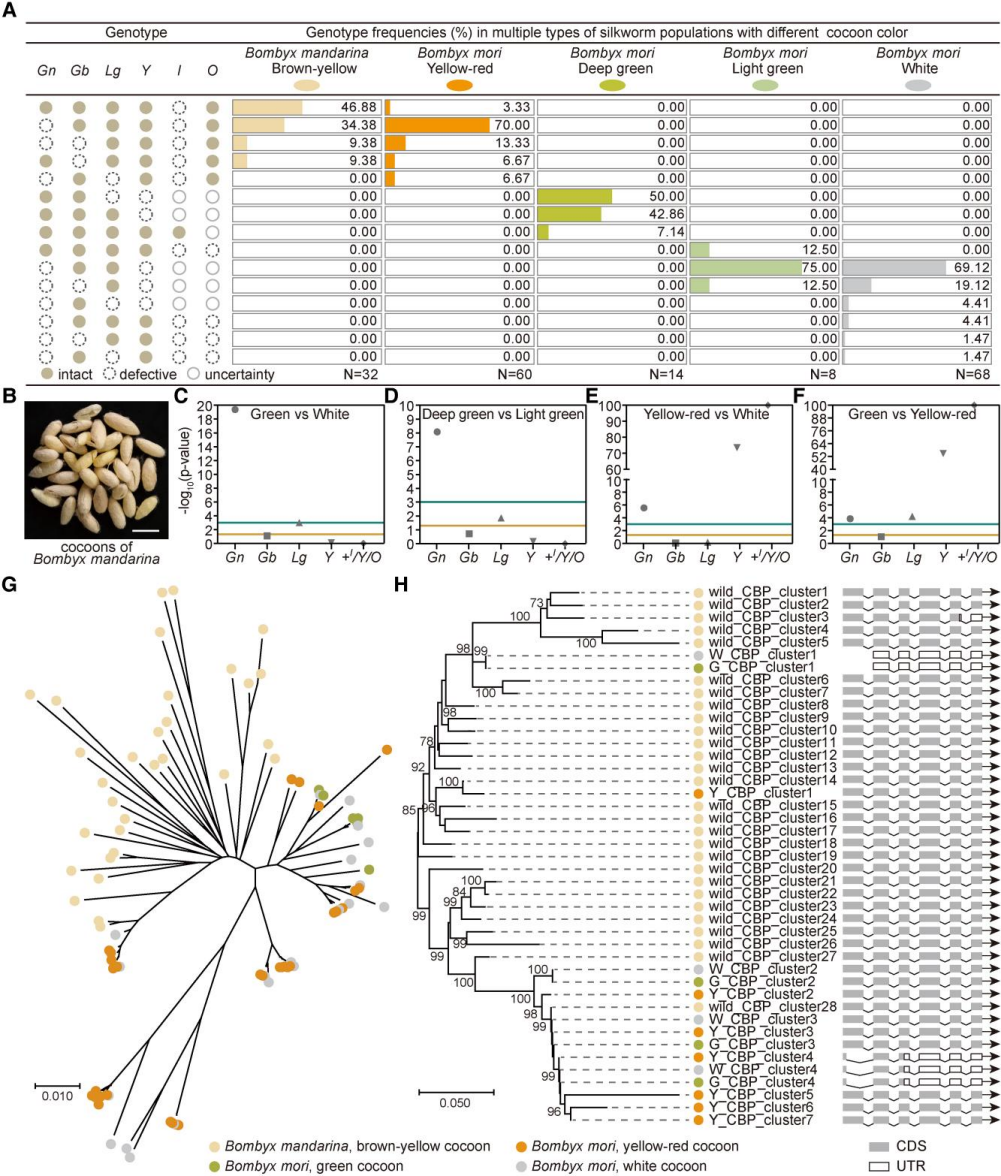
Formation and evolution of multicolored silkworm cocoons. (*A*) The combinations of cocoon color-related loci create multicolored cocoons. *Gn* is recorded as “defective” when any member of *Gn_Strs* is defective. *O* is recorded as “intact” when any of the *C*, *F*, *Rc*, or *Pk* exist. (*B*) Cocoons of *B. mandarina.* Scale bar, 2 cm. (*C*–*F*) Association of each locus with cocoon color. In the one-way ANOVA, the genotype of “intact” and “defective” were assigned a value of 1 and 0, respectively. Wild silkworms were classified into the group “yellow-red.” If the *P*-value of one-way ANOVA was 0, then -log_10_ (*P*-value) was recorded as 100. (*G* and *H*) Phylogenetic analysis of *Gn_Strs* and *CBP*. The sequences in each population were deredundant with the CD-HIT online tool. The percentages of replicate trees in the bootstrap test (1,000 replicates) are shown next to the branches of CBP's phylogenetic tree, only values greater than 70% are displayed.

The refinement of the molecular basis underlying flavonoids-based green cocoon and the construction of high-quality silkworm pan-genome sequencing data in our team (Tong et al. 2022) provides an opportunity to systematically explore the formation of polymorphic cocoon colors and further elucidate their evolutionary processes. First, to reveal the cocoon color differentiation accompanied by different combinations of cocoon color-related loci, we genotyped cocoon color-related loci (supplementary table S10, Supplementary Material online) in multiple wild and domestic silkworms and analyzed the association between each locus and different cocoon colors. As already reported, *Gb*, whose functional protein BmUGT10286 catalyzes the glycosylation modification of the quercetin 5-O position (Daimon et al. 2010), is essential for green cocoon formation. About 95.45% (21/22) of the green cocoon strains had intact *BmUGT10286*, except for one with a pale green cocoon (fig. 8*A*, supplementary table S6, Supplementary Material online). However, *BmUGT10286* was also intact in more than 80% of the strains in nongreen cocoon populations, including wild silkworms with brown-yellow cocoons and domestic silkworms with white and yellow-red cocoons (supplementary fig. S10, Supplementary Material online). The distribution of *Lg* among different populations is also similar (supplementary fig. S10, Supplementary Material online). These results clarified why only a single locus, *Gn* on chromosome 27, was detected in GWAS, whereas evidence from both classical and molecular genetic studies demonstrated that multiple loci distributed on different chromosomes were associated with the green cocoon (Xiang 2005). *Gn* differs from these loci in that it clearly distinguished between white and green cocoon populations, which is intact in most of the strains with green cocoons and defective in strains with white cocoons (fig. 8*A*, supplementary table S6, Supplementary Material online); thus, it exhibited the most significant association signal in GWAS and the one-way ANOVA (figs. 2*A* and 8*C*).

Classical genetic studies showed that the coexistence of *+^I^*, *Y*, and at least one of the cocoon color-related loci (*C*, *F*, *Pk*, and *Rc*, collectively referred to as *O* henceforward) is essential for forming yellow-red cocoons; this was also supported by our data at the molecular level (fig. 8*A*, supplementary table S6, Supplementary Material online). What caught our attention was that when the yellow-red cocoon-related loci *Y*, *+^I^*, *O*, and the green cocoon-related loci *Gn* and *Gb* coexisted, the cocoons appeared yellow-red instead of green both in wild and domestic silkworms (fig. 8*A*and*B*). Then, we treated *Y*, *+^I^*, and *O* as a whole and marked them as +*^I^*/*Y*/*O*. When they coexist, +*^I^*/*Y*/*O* was assigned a value of 1, otherwise 0, and then a one-way ANOVA was performed. It was found that +*^I^*/*Y*/*O* was completely distinguished between green and yellow-red cocoon strains with a *P* value of 0 (fig. 8*F*). In particular, *Y* was defective in most of the strains with green cocoons and exhibited a high correlation signal between strains with green and yellow-red cocoons (-log10 [*P*-value] = 54.73; fig. 8*A*and*F*). The same was applied to the strains with yellow-red and white cocoons (fig. 8*A*and*E*).

Then, to reveal the evolutionary process of cocoon color, phylogenetic analyses were performed on the genes responsible for *Y* and *Gn*, which were highly associated with cocoon color (fig. 8*C*–*F*). More than half of the wild silkworms possessed intact *Gn_Strs*, the genes responsible for *Gn*; this suggested that the *Gn_Strs* in domestic silkworms were initially derived from wild silkworms. Phylogenetic analysis of the nucleotide sequence in the *Gn_Strs* genomic region indicated high sequence similarity among domestic silkworms with different cocoon colors (fig. 8*G*). Moreover, the *Gn_Strs* in domestic silkworms were distinguished from those of wild silkworms (fig. 8*G*). This suggested that the *Gn_Strs* in domestic silkworms that build different colored cocoons were not separately acquired from wild silkworms but shared a common domestic silkworm ancestor. Then, the evolutionary process of *CBP* responsible for *Y* was analyzed. There was reported copy number variation in CBP among different populations (Sakudoh et al. 2011). Here, at least one intact CBP was identified in all investigated wild silkworms and domestic silkworms building yellow-red cocoons. A few of these strains carried both defective and intact CBP copies (supplementary table S11, Supplementary Material online). In the populations with white and green cocoons, there were mainly two types of defective CBP: complete deletion (W/G_CBP_cluster1) and partial deletion (W/G_CBP_cluster4) of the first exon of CBP (fig. 8*H*). Phylogenetic analysis showed that these two types of defective CBP had high sequence similarity with the intact CBP copies in wild silkworms and the imperfect CBP copies in domestic silkworm strains with yellow-red cocoons (Y_CBP_cluster4), respectively (fig. 8*H*). There are also a few strains building green and white cocoons containing defective *O* and intact CBP (W/G_CBP_cluster2, W/G_CBP_cluster3; fig. 8*A*and*H*), which have higher sequence similarity with *CBP* copies in domestic silkworms constructing yellow-red cocoons than in wild silkworms (fig. 8*H*).

**Fig. 9.**
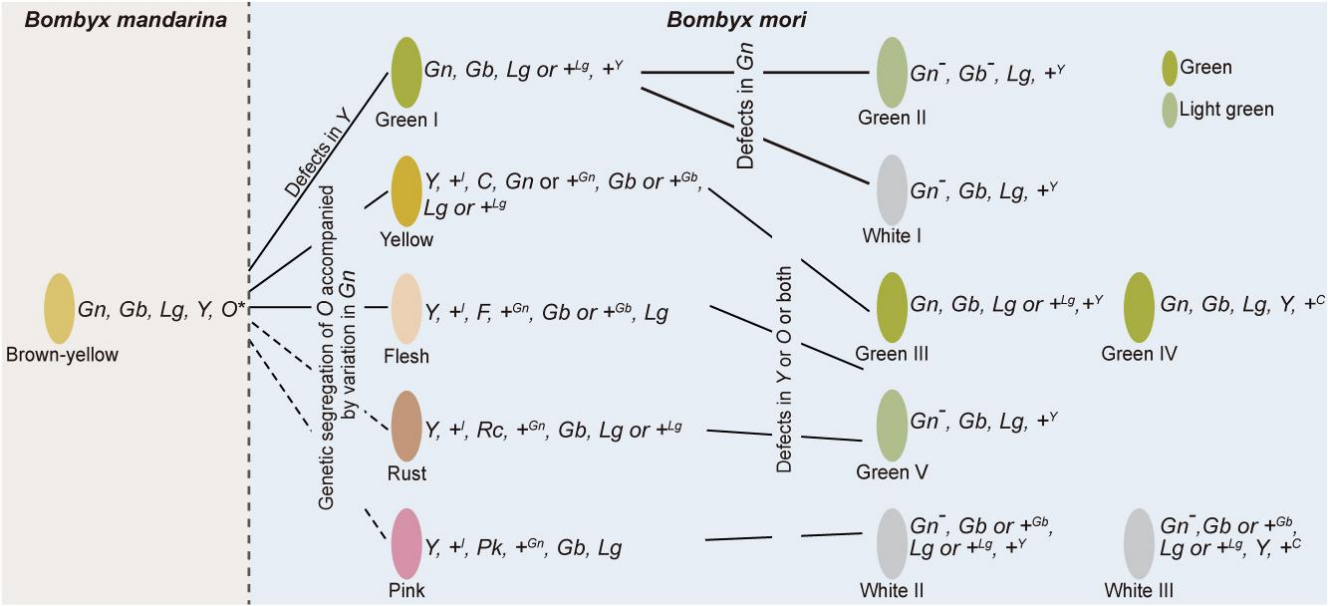
Proposed model for the evolutionary process of cocoon color. The long ovals filled with different colors refer to cocoons in different colors; their corresponding genotypes are shown next to the right. *F and *C* coexist in most of the wild silkworm strains (25/32) by investigating the genome sequences of their functional genes, and the genotypes of *Rc* and *Pk* are indeterminate.

Integrating these results, we propose a model for the formation and evolution of cocoon color. There are five core points. 1) The cocoon color-related genes identified in domestic silkworms derived initially from the wild silkworm. 2) The yellow-red cocoon is probably an ancient trait, with the *Y* in the domestic silkworm derived from the wild silkworm, and the formation of different yellow-red cocoons involves the genetic segregation of *O*. 3) The yellow-red cocoon has an epistatic effect on the green cocoon. 4) Mutations leading to the knockout of *Y* or *O*, especially the *Y*, are vital nodes in the emergence of green and white cocoons. 5) there are a few strains with green and white cocoons containing defective *O* and intact *CBP*; these green and white cocoons may be caused by the sequence variations leading to defects in *O* in the domestic silkworm strains building yellow-red cocoons. (fig. 9).

## Discussion

The results of this work uncovered two crucial issues regarding cocoon coloration. First, the molecular basis of the flavonoids-based green cocoons was refined: a cluster of Bombycidae-specific sugar transporter duplicates with complementary temporal expression synergistically facilitated the uptake of flavonoids, thus determining the green cocoon. Second, the formation and evolution of polymorphic cocoon colors were deciphered, and a corresponding model was proposed. To our knowledge, this is the first identification of membrane transporters involved in the uptake of flavonoids in silkworms and is also a discovery of the mechanism underlying biological coloration. It is also the first proposal for the evolution process of cocoon color.

### The Possible Role of Gn_Strs in Forming the Green Cocoon

**Fig. 10.**
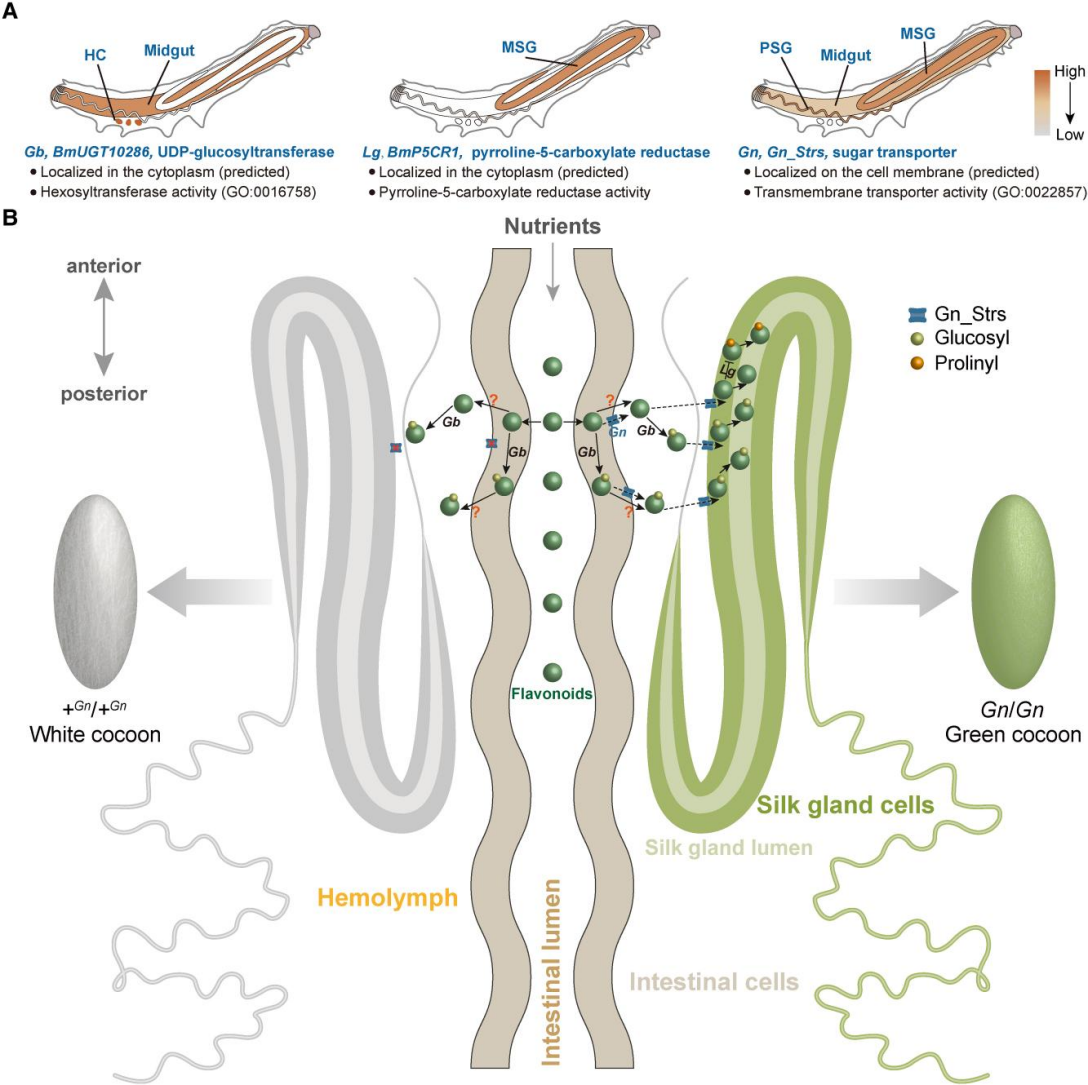
Proposed working model for identified green cocoon-related genes. (*A*) Spatial expression patterns of identified green cocoon-related genes. (*B*) Role of BmUGT10286, BmP5CR1, and Gn_Strs in forming green cocoons.

Different from BmUGT10286 and BmP5CR1, which are located in the cytoplasm and catalyze the chemical modification of flavonoids (Daimon et al. 2010; Hirayama et al. 2018), the Gn_Strs are located in the membrane and belong to the MFS that mediate transmembrane transport of a wide range of substrates (Yan 2015). Classical genetic evidence in silkworms suggested that regardless of the essential roles of *Gb* and *Lg* in the metabolic modification of flavonoids, neither can cause green cocoons independently, and their cooperation with proteins responsible for flavonoids transport is necessary (Xiang 2005). Echoing this view: our data suggested that the coexistence of *Gn_Strs* and *BmUGT10286* is required for forming green cocoons, especially deep green cocoons. Gn_Strs are homologous to the facilitated glucose transporters (GLUTs) in *Homo sapiens* (supplementary fig. S11, Supplementary Material online). Studies in mammalian cells have shown that flavonoids can inhibit the uptake of substrates mediated by several members of GLUTs, such as the GLUT1, GLUT2, GLUT5, GLUT7, and GLUT8 (Chen et al. 2007; Kwon et al. 2007; Corpe et al. 2013; Gauer et al. 2018; Ojelabi et al. 2018). Hence, flavonoids are speculated to be potential competitive substrates of GLUTs (Chen et al. 2007; Passamonti et al. 2009). However, this view has been controversial due to the limitations of in vitro models and the lack of valid evidence. Here, it was supported by our findings based on animal experiments that the knockout of GLUT homologs in the silkworm, Gn_Strs, resulted in a substantial reduction of flavonoids content in the SG and cocoons. Synthesizing these results, we propose a model for the cooperation of BmP5CR1, BmUGT10286, and Gn_Strs in forming green cocoons: the former two participate in the metabolic modification of flavonoids, glycosylation in MG and HC (Daimon et al. 2010), prolinyl modification in SG, respectively (Hirayama et al. 2018); the latter is accountable for the transport of flavonoids (fig. 10). Deficiencies in either of these roles would lead to defects in the green cocoon phenotype.

### Evolution of *Gn_Str*_cluster

The daughter copy functions are similar to the parental copy when a gene has just completed duplication. Such functionally redundant genes usually cannot be stably retained in the genome because they are invisible to natural selection unless they undergo rapid functional evolution, such as neofunctionalization, subfunctionalization, or both (Qian et al. 2010). There are more than 150 *Strs* in the silkworm, likely due to duplication, and most of them are tissue-specifically expressed (supplementary fig. S12, Supplementary Material online); this is evidence that they undergo subfunctionalization. Nevertheless, the simultaneous retention of so many *Strs* in the silkworm genome remains incomprehensible. For example, nearly 20 *Strs* specifically expressed SG (supplementary fig. S12, Supplementary Material online); how do they perform a good division of labor or maintain functional redundancy? In this study, the genetic dissection of *Gn_Str*_cluster gives us an excellent example to understand this question.

*Gn_Str*_cluster was likely produced by tandem gene duplication with *BmStrGn1* or *BmStrGn2* that were conserved in Lepidoptera as the parental copy. Especially, BmStrGn2 has higher sequence similarity with other Gn_Str_cluster members (fig. 7*D*, supplementary fig. S9, Supplementary Material online). Noticeably, the relative order of *BmStrGn1* and *BmStrGn2* in silkworms differs from that of most other species (fig. 7*A*). Therefore, the rearrangement of these two genes, which may involve broken and fragmented rearrangements within the locus, maybe the first step in *Gn_Str*_cluster formation. And the tandem duplication with *BmStrGn2* as a parental copy after the rearrangement of *BmStrGn1* and *BmStrGn2* may be a more feasible molecular mechanism for the emergence of *Gn_Str*_cluster. The new members of *Gn_Str*_cluster, *Gn_Strs*, emerged accompanying the functional evolution that firstly manifested in their spatial expression pattern: the putative parental copy of *Gn_Strs*, *BmStrGn2*, was primarily expressed in MG (fig. 7*D*). BmStrGn7 had a high sequence identity with BmStrGn2 and exhibited broad expression with the highest expression level in the MG, followed by the SG (fig. 7*D*); it may play the role of the secondary parental copy of *BmStrGn3*, *BmStrGn4*, *BmStrGn5*, and *BmStrGn6. BmStrGn3* to *BmStrGn6* precisely duplicated in the Bombycidae (fig. 6) and evolved specialized spatial expression patterns, expressed only in silk glands (fig. 7*D*); this implies their specific or novel functions in the silkworm. Further studies proved that they possess similar molecular functions in participating in the uptake of flavonoids and contributing to the green cocoon (fig. 5). It should be noted that they are not redundant in the strict sense. They achieved dosage balance by dosage-sharing (Qian et al. 2010; Lan and Pritchard 2016). In more detail, *Gn_Strs* show a complementary temporal expression; when one *Gn_Str* was downregulated, the other one was raised (fig. 7*E*); this is a manifestation of *Gn_Strs* undergoing subfunctionalization (Innan and Kondrashov 2010). In summary, *Gn_Strs* underwent neofunctionalization accompanied by subfunctionalization in Bombycidae; this gene functional evolution process is similar to that of the subneofunctionalization model (He and Zhang 2005). Such functional evolution of *Gn_Strs* may be crucial for their simultaneous retention in the genome. Nonetheless, the substrate specificity of Gn_Strs remains to be further investigated.

Due to flavonoids or carotenoids, the natural-colored cocoons had more robust antibacterial, antiultraviolet, and antioxidation properties than the white cocoons (Kurioka et al. 1999; Kurioka and Yamazaki 2002; Xu et al. 2003; Daimon et al. 2010; Wang et al. 2020). Regardless of the domestic silkworms living indoors, the *Gn_Strs* specifically duplicated in the Bombycidae may provide a potential advantage for wild silkworms under natural selection pressure: even under the epistasis of the yellow-red cocoon, green cocoon-associated genes still cause small amounts of flavonoids to accumulate in the cocoons (Zhang et al. 2019), which may be beneficial for the survival of wild silkworms. Thus, the evolutionary process of *Gn_Str*_cluster highlights the crucial role of gene duplication followed by functional diversification, an important mechanism of genome evolution and an essential source of new genetic functions, in acquiring genetic traits for environmental adaptation (Long et al. 2003, 2013; Chen et al. 2013).

### The Evolution Process of Cocoon Color from Homogenization to Diversification during Domestication

To construct a polymorphic coloring landscape of silkworm cocoons and clarify their evolution process, we investigated the identified cocoon color-related loci in various silkworm populations building different colored cocoons and performed phylogenetic analysis on loci that exhibited highly correlated signals with cocoon color.

All the cocoon color-related loci identified in the domestic silkworm exist in the wild silkworms, including the green loci *Gn*, *Gb*, *Lg*, and the yellow-red loci *Y*, *O* (*C* and *F* exist, *Rc* and *Pk* are uncertain; fig. 8*A*, supplementary table S6, Supplementary Material online). It is striking that the genotypes of cocoon color-related loci in domestic silkworms with yellow-red cocoons were similar to those of wild silkworms (fig. 8*A*). Therefore, we consider that the yellow-red cocoon is an ancient trait, with the *Y* in the domestic silkworm derived from the wild silkworm, and the formation of different yellow-red cocoons involves the genetic segregation of O that is also derived initially from the wild silkworm (fig. 9).

*Y* and *O* coexist in all investigated wild silkworms; about half of these also contain *Gn* and *Gb* (supplementary table S6, Supplementary Material online), and their cocoons are yellow rather than green (fig. 8*B*). Several strains containing *Y*, *O*, *Gn*, and *Gb* were still identified in *B. mori*, such as BomL122 and BomM335 (supplementary table S6, Supplementary Material online), and their cocoons were also golden yellow instead of green. Furthermore, in all silkworm strains with a green cocoon, one or both of *Y* and *O* is absent, or there was an *I* that inhibited *Y*. All the evidence suggested that the yellow-red cocoon is epistatic to the green cocoon. There are two essentials to form green cocoons: the presence of green cocoon-related loci, *Gn*, *Gb*, etc., and the presence of *I* or the absence of *Y* or *O*. Especially the *Y*, which was deficient in most (20/22) strains with a green cocoon (fig. 8*A*, supplementary table S6, Supplementary Material online).

Among all the identified cocoon color-related loci (supplementary table S10, Supplementary Material online), *Gn* and *Y* exhibited high association signals with cocoon color (fig. 8*C*–*F*). Therefore, phylogenetic analyses were performed on their responsible genes, *Gn_Strs* and *CBP*. Phylogenetic analysis of *CBP* revealed that the W/G_CBP_cluster1, a major defective CBP in the populations with green and white cocoons, had high sequence similarity with the CBP in wild silkworms (fig. 8*H*). Another major defective CBP, W/G_CBP_cluster4, had extremely high sequence similarity with the Y_CBP_cluster4 present in a few numbers of strains with yellow-red cocoons. Based on these results, we propose two primary sources of white and green cocoons: one is the defect of CBP at the beginning of silkworm domestication (green I with W/G_CBP_cluster1). The other is the defect of CBP after the differentiation of the yellow-red cocoons (green III, green V, and white II with W/G _CBP_cluster4); whether these strains inherit the *+^Gn^* or *Gn* derived from the silkworms with yellow-red cocoons determines whether the cocoon is green, light green, or white. A few strains with green and white cocoons possessed complete CBP and defective O (green IV and white III with W/G_CBP_cluster2 and W/G_CBP_cluster3); this may be the result of defective variants following the genetic segregation of *O*. Defects in either *Y* or *O* lead to the removal of the epistatic effect of the yellow cocoons on the green and white cocoons. These conclusions are also supported by the results of phylogenetic analysis on *Gn*: the *Gn_Strs* genomic region of most of the strains with green and white cocoons have high sequence similarity with the strains building yellow-red cocoons but not the wild silkworm. Furthermore, the emergence of a few white and pale green cocoons does not go through the stage of yellow-red cocoons (white I, green II). They contained the same type of *CBP* as green I, W/G_CBP_cluster1. Their phenotypes may result from defects in *Gn* in green I (fig. 9). Overall, genetic segregation, recombination, and mutation of the cocoon color-related loci initially derived from the wild silkworm shaped the various cocoon color in domestic silkworms. These findings reflect the great potential of wild animals to evolve diverse phenotypes, which may be an essential safeguard for them to cope with environmental changes. This part of the work gives us a deeper understanding of the rapid phenotypic innovation from simple to diverse during domestication.

## Materials and Methods

### Photographing of SG and Cocoons under Natural and UV Light

The cocoon shells were cut into fragments about 1.5 cm in diameter to let the UV emitted from the underneath light source pass through. The SG dissected from caterpillars were placed in transparent Petri dishes. Then, SG and cocoon fragments were imaged by the UV transmission mode of the gel imaging system (GenoSens 1880S, Clinx Science Instruments Co., Ltd.). The exposure time is indicated in the corresponding illustration.

### Silkworm Strains

All the silkworm strains were obtained from the Silkworm Gene Bank of Southwest University, Chongqing, China.

872B and N100 are a group of near-isogenic lines against *Gn*. In the construction of N100, G200 was used as the donor parent of *Gn*. First, G200 was crossed with 872B, their F_1_ generation was backcrossed with 872B, and then the individuals building green cocoons were selected in the BC_1_ generation and recrossed with 872B. Repeat ten times like this, and finally, the progeny building green cocoons were self-crossed several times until a pure line for the *Gn* locus was obtained, named N100; after this manipulation, most of the genome of N100 was derived from 872B except for the *Gn* locus and its linked sequences.

### Genome-Wide Association Analysis

The SNPs of 111 silkworm strains were used for GWAS. Among these, 60 and 51 strains build white and green cocoons, respectively (supplementary table S2, Supplementary Material online). SNPs with MAF < 5% were filtered out. GWAS was performed using an additive logistic regression model in PLINK (Purcell et al. 2007).

### Positional Cloning of *Gn*

The mating schemes to obtain the population for positional cloning of *Gn*: the female and male F_1_ heterozygotes of G200 and 872B were backcrossed with 872B to bring BC_1_F and BC_1_M populations, respectively (supplementary fig. S1, Supplementary Material online). Ten and ten individuals building white and green cocoons in BC_1_F, respectively, and 1,035 individuals building white cocoons in BC_1_M were used in the linkage analysis between *Gn* and 17 polymorphic molecular markers on chromosome 27. The primer sequences for positional cloning of *Gn* are shown in supplementary table S3, Supplementary Material online. The genomic DNA of each individual was extracted using the phenol-chloroform/proteinase-K method.

### Annotation of Genes in *Gn* Locus

The gene annotation information was retrieved from the KAIKObase (https://sgp.dna.affrc.go.jp/KAIKObase/). The gene information of *Strs* was updated by a comprehensive analysis of SilkBase (http://silkbase.ab.a.u-tokyo.ac.jp/cgi-bin/index.cgi), SilkDB 3.0 (https://silkdb.bioinfotoolkits.net/main/species-info/-1), and SilkTransDB (Li et al. 2012) (http://124.17.27.136/gbrowse2/). Ultimately, the physical location information of all genes was subject to the SilkBase.

Domain analysis and functional annotation of proteins were carried out with the online tools of Pfam (http://pfam.xfam.org/), NCBI CD-search (Conserved Domain Search, https://www.ncbi.nlm.nih.gov/Structure/cdd/wrpsb.cgi), UniProt (https://www.uniprot.org/), and NCBI blast (https://blast.ncbi.nlm.nih.gov/Blast.cgi). The transmembrane helices of Strs were analyzed by the HMMTOP online tool (Tusnady and Simon 2001).

### RNA Extraction, Acquisition of cDNA and qRT-PCR

The total RNA of each sample was extracted using the TransZol Up Plus RNA Kit (ER501, TransGen Biotech, China). The PrimeScript RT reagent kit with gDNA Eraser (RR047, Takara, Japan) was used for the reverse transcription. The reaction system and methods used in qRT-PCR were consistent with the published data (Lu et al. 2016). Relative gene expression levels were normalized to the silkworm housekeeping gene *eIF*4A (eukaryotic translation initiation factor 4A, *BMgn003186*). Primer sequences for qRT-PCR are listed in supplementary table S5, Supplementary Material online.

### CRISPR/Cas9-Mediated Gene Knockout

The genes’ particular sgRNA target sites were screened using the online tool CRISPRdirect (http://crispr.dbcls.jp/). Two sgRNA target sites were designed for each gene to ensure gene editing efficiency. The sgRNA target sequences (20 nt + PAM) are listed in supplementary table S7, Supplementary Material online. Knockout lines 1, 2, 4, and 5 were obtained by homozygous screening of the crossing-over individuals that expressed sgRNA-Cas9. The methods of vector construction, transgenic line acquisition, and homozygous screening were referred to in the previously published studies (Gai et al. 2020). The knockout line 3 was obtained by injecting the mixture of Cas9 protein and sgRNA and then carrying out homozygous screening. SgRNA was synthesized using T7 RiboMAX Express Large-Scale RNA Production System (P1300, Promega, USA) and then diluted to 1,000 ng/μl. Nine microliters of sgRNA were added to 1 μl of TrueCut Cas9 Protein v2 (A36499, Invitrogen, USA) and gently mixed. The mixture was incubated at 37 °C for 15 min and then used for microinjection, where the amount of sgRNA injected into each egg was 8–10 ng. The hatched caterpillars and the offspring were raised, and the adult moths were subjected to molecular detection to determine the stable genetic line of *BmStrGn5* to *BmStrGn6*_KO.

### Determination of Total Flavonoids in SG and Cocoons

The SG was ground into a powder with liquid nitrogen and freeze-dried in a freeze-dryer for 24 h. Cocoons were cut into small pieces with a diameter of about 0.5 cm. Total flavonoids were extracted from 0.1 g cocoon shell samples or SG powder with 40% ethanol solution; the ratio of solid to liquid was 1:20, and ultrasonic extraction of flavonoids was carried out for 20 min at 600 W. The supernatant was then separated, and the residue was extracted again. The two extracts were mixed and stored at 4 °C. Total flavonoids content was measured as rutin equivalents using a validated UV spectrophotometric method (Jia et al. 1999). The rutin was used as the standard for constructing the calibration curve.

### Construction of Phylogenetic Trees

Multiple sequence alignment of protein sequences corresponding to figures 6 and 7*D*, supplementary figures S7 and S11, Supplementary Material online was carried out in Muscle (Edgar 2004). Multiple sequence alignment of nucleotide sequences corresponding to figures 4*B* and 8*G*and*H* was carried out in MAFFT (Katoh et al. 2002) and Muscle(Edgar 2004), respectively. Then, the phylogenetic trees of figures 4*B*, 6, 7*D*, and 8*G*and*H*, and supplementary figure S11, Supplementary Material online were constructed using the Mega software and Neighbor-joining method with 1,000 bootstrap replications (Kumar et al. 2018). The phylogenetic trees corresponding to supplementary figure S7, Supplementary Material online were not examined due to the overloaded computing.

### Identification of all Strs Encoded by the Genome of Insects and Humans

The Sugar_tr (PF00083) Hidden Markov model profile was downloaded from Pfam. The genome-wide protein sequences of insects and *H. sapiens* were downloaded from InsectBase 2.0 (http://v2.insect-genome.com/) and CCDS database (http://www.ncbi.nlm.nih.gov/CCDS/), respectively. The Hmmsearch program of HMMER 3.0 (Finn et al. 2011) software was used to search Strs. Redundant sequences in each species were removed with the CD-HIT online tool (Fu et al. 2012).

### Genotype Investigation of Cocoon Color-Related Loci in Multiple Strains

The genotypes of *Gn*, *Gn*, *Lg*, and *Y* that have been elucidated were determined by sequence investigation for their responsible genes based on the published data (Sakudoh et al. 2007; Daimon et al. 2010; Hirayama et al. 2018). The genotype of *I* and *O* in domestic silkworms was inferred from classical genetic studies. The genotype of *O* in wild silkworms, explicitly referring to the elucidated *F* and *C*, was preliminarily determined by sequence investigation for their responsible genes. The genotypes of *Rc* and *Pk* in wild silkworms are unknown due to a lack of evidence from molecular and classical genetics.

### Spatial Expression Clustering of all *Strs* in the Silkworm Genome

The expression data of *Strs* within the *Gn* locus were obtained by qRT-PCR, and the expression data of other genes were downloaded from the database of SilkDB3.0. Gene expression clustering and charting were done with the TBtools (Chen et al. 2020).

## Supplementary Material

msad017_Supplementary_Data
