# Supplementary material for: Deciphering the Genetic Basis of Silkworm Cocoon Colors Provides New Insights into Biological Coloration and Phenotypic Diversification
Source: Mol Biol Evol. 2023 Jan 31;40(2):msad017. doi: 10.1093/molbev/msad017 (PMC9937047; doi:10.1093/molbev/msad017)
Supplement: msad017_Supplementary_Data [file msad017_supplementary_data.zip › Supplementary figures.pdf]

Supplementary Figures and Legends

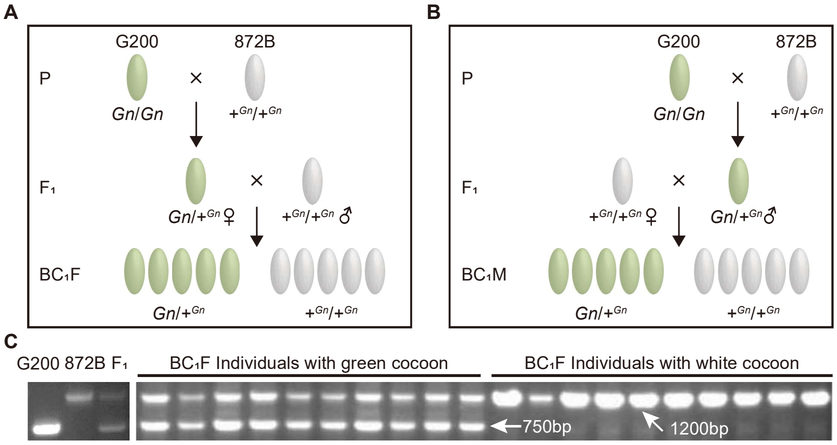

Figure S1 Positional cloning of *Gn*. (A) The mating scheme to obtain the population for verifying the chromosome where *Gn* is located. (B) The mating procedure to get the population for positional cloning of *Gn*. (C) Genotyping of the representative polymorphic molecular marker, M11, in the BC_1F population.

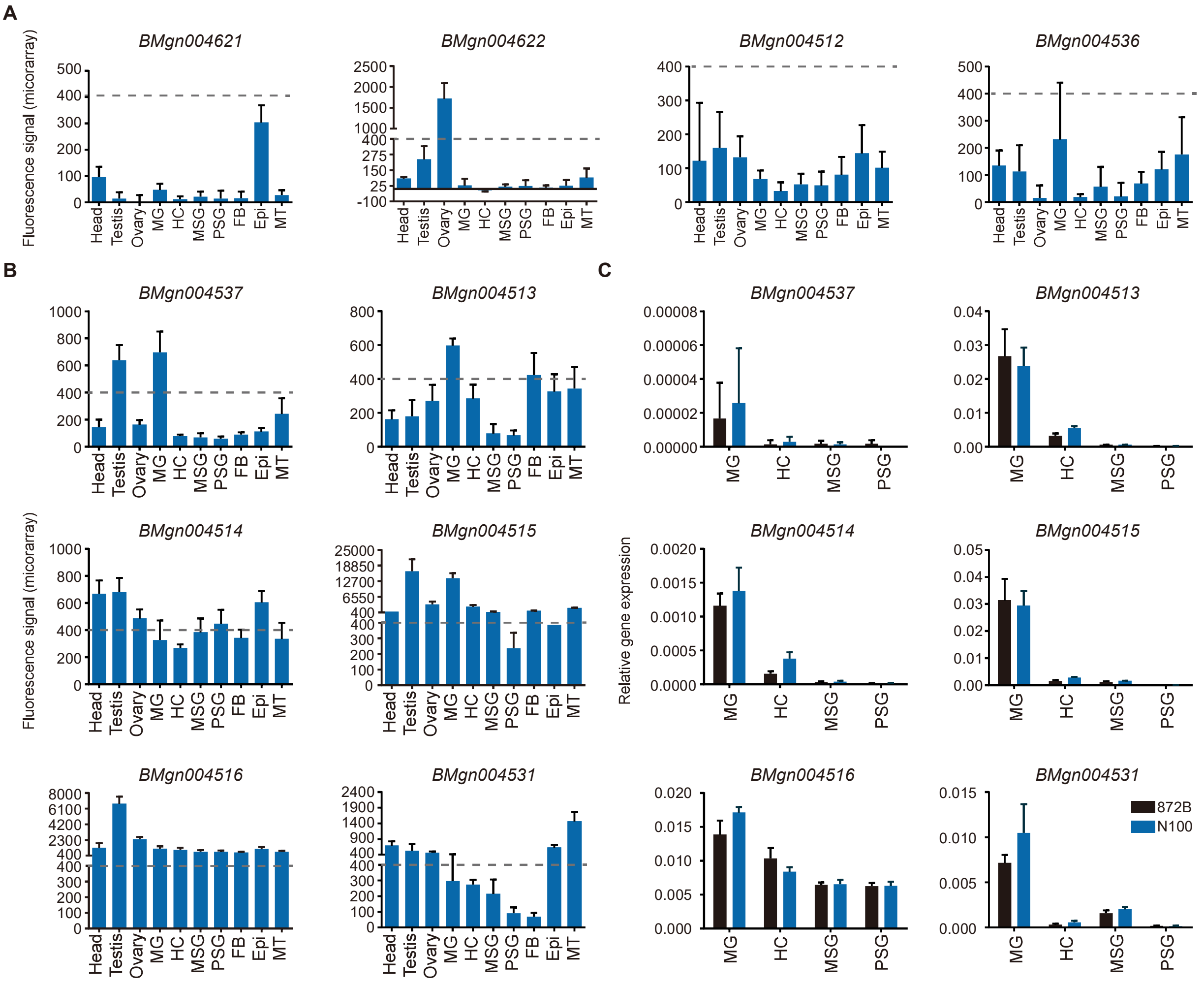

Figure S2 Expression of non-*Str* genes in the *Gn* locus. (A) and (B) Spatial expression of non-*Str* genes. Gene expression information was obtained from the published genome-wide gene expression profile microarray data of Dazao (green cocoon, *Gn*/*Gn*) caterpillars on the third day of the fifth instar. The genes in (B) were expressed in the MG, HC, or SG, while the genes in (A) were not. FB, fat body; Epi, epidermis; MT, malpighian tubules. The gray dotted lines indicate the threshold, 400. Error bars represent mean ± SD (n = 4 to 8 biological replicates). (C) Expression of genes in the MG, HC, and SG of N100 and 872B caterpillars on the third day of the fifth instar. (n = 3 biological replicates).

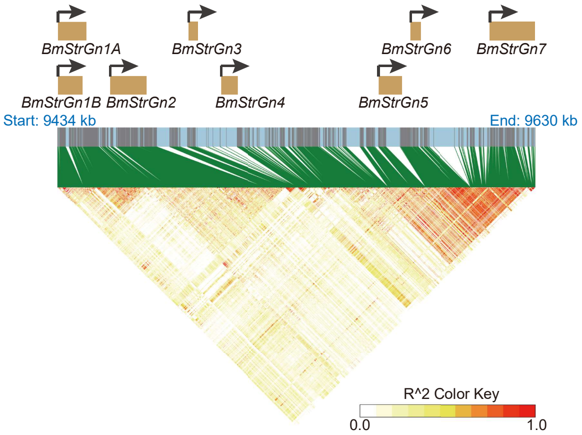

Figure S3 Heat map of linkage disequilibrium in the genomic region where the *Gn_Str*_cluster is located.

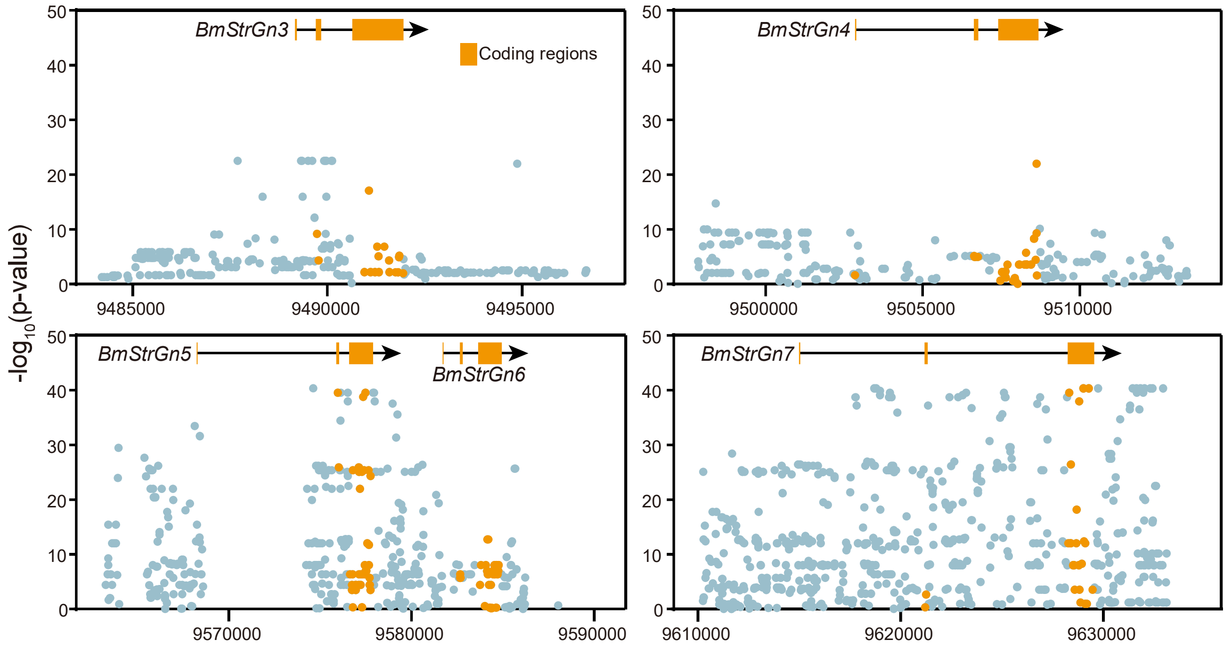

Figure S4 Regional Manhattan plot of each *Gn_Str*. The orange dots represent SNPs located in the *Gn_Str* coding regions.

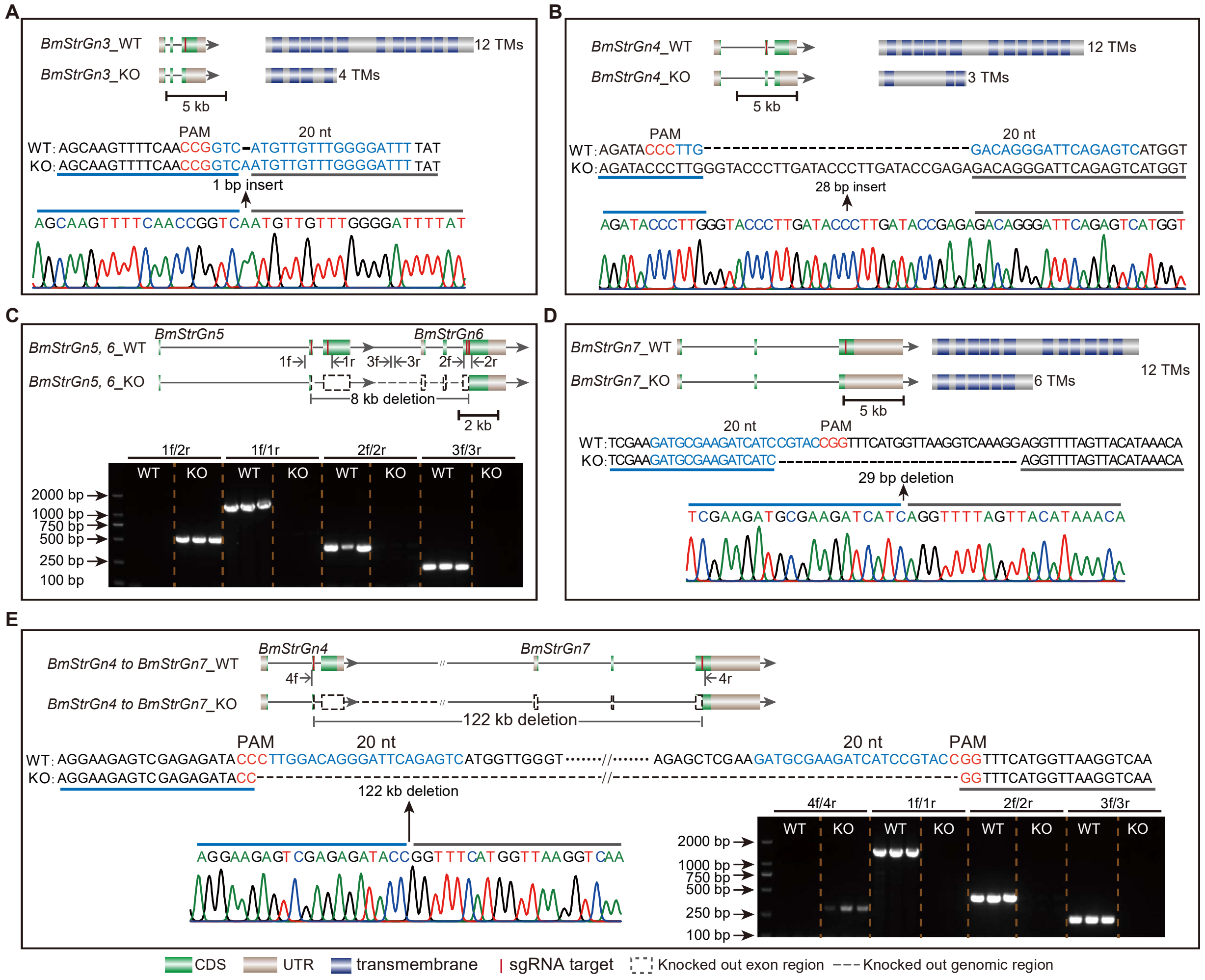

Figure S5 CRISPR/Cas9 mediated *Gn_Strs* knockout. (A) to (E) Schematic diagrams of gene structure variation caused by CRISPR/Cas9-mediated gene knockout and PCR products sequencing or electrophoresis of sgRNA target sites of *BmStrGn3*, *BmStrGn4*, *BmStrGn5* to *BmStrGn6, BmStrGn7*, and *BmStrGn4* to *BmStrGn7* knockout lines, respectively.

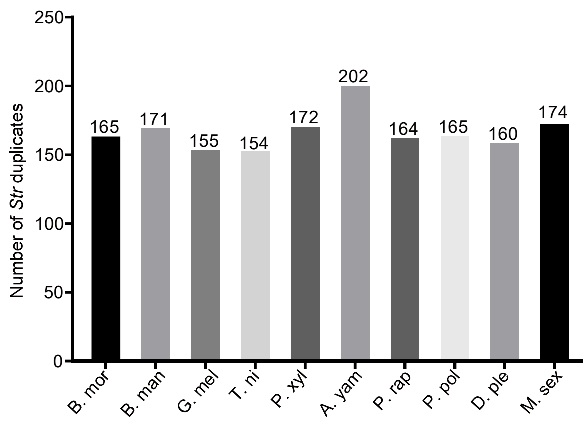

Figure S6 Number of Str duplicates encoded by the genome of ten Lepidoptera insects. *B. mor*, *Bombyx mori*; *B. man*, *Bombyx mandarina*; *P. rap*, *Pieris rapae*; *P. xyl*, *Plutella xylostella*; *D. ple*, *Danaus plexippus*; *M. sex*, *Manduca sexta*; *A. yam*, *Antheraea yamamai*; *G. mel*, *Galleria mellonella*; *P. pol*, *Papilio polytes*; *T. ni*, *Trichoplusia ni*.

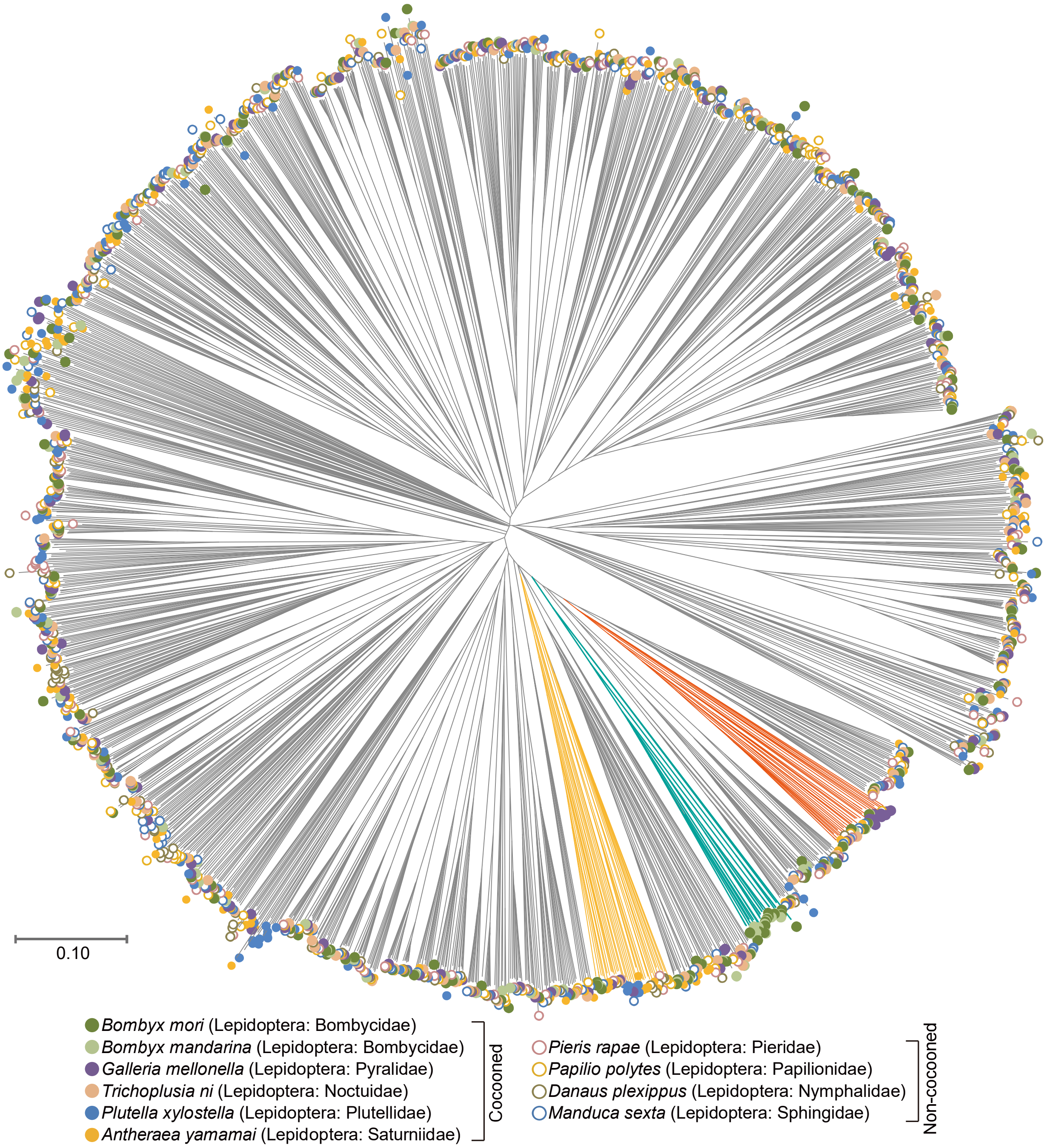

Figure S7 Phylogenetic analysis of all Strs encoded by the genome of ten Lepidoptera insects. The subtrees in which BmStrGn1, BmStrGn3 to BmStrGn6, BmStrGn2 and BmStrGn7 are located are highlighted in orange, green, and orange-red, respectively.

```
BmStrGn1A : -------MKPFLKQTWAVSAVLINMVGQGILLSYTTSLLPALQAPDSPIPTDLDTASWLSSSVGISGIPGFFMSSTLMEAYGRKVAHFIIMMPGLIGW : 91
BmStrGn1B : -------MKPFLKQTWAVSAVLINMVGQGILLSYTTSLLPALQAPDSPIPTDLDTASWLSSSVGISGIPGFFMSSTLMEAYGRKVAHFIIMMPGLIGW : 91
BmStrGn2  : -MKKNQWITPFKKQCFVTAGVCLNMASHGLVMGFSAILVPQLQQPDSPIPVDYSTGSWIASIPGFALVVGNFIVPTIMAKYGRRTANLISIAPMITGW : 97
BmStrGn3  : MPRRRQWMTPFIKQCFVTSGVTLNMLTHGFMYGFNTGLFAQLRKTKE-IQLDLELESWLASSTSLSFIVGSLFSSIFMDRFGRRPAFIIASATMIISW : 97
BmStrGn4  : MSGTGRWTTPFLKQCFAVSSVALNVMSYGMVFGFNASLFSDLRKSRE-IPLDRDSESWLASLIGITTLIGCFSTSIIIDTIGRRPALLISSTLMVSGW : 97
BmStrGn5  : MCS----VTPFTKQCFAIASVIVNMMSYGMCIGFNASLFSELKKTGE-IPLDRDSESWLASLIGITTLIGALLTSFIIDSIGRRPAVITSSVLMVSGW : 93
BmStrGn6  : MCRIRRWKTPFLKQCLVISSVTFNMISYGFAFGFNASLITSLRKTNE-IPLNRDSESWLASVLGLFIFIGSIITCIIMKYIGRRFSFLLSSIIIICSW : 97
BmStrGn7  : -MVLEKWITPFKRQCFVVAGVSLNMSQLGMVFGYAAILVPQLRKSDSLIPIDEASESWVAALPGFALVLGNLVVPPVMAKFGRRIANIISIVIVLTGW : 97

BmStrGn1A : LLIYFGNNIPVLMIGRILGGMSAGGTVALGAIVIGEYSSPKYRGMYLNMKTASVCLGGMLVHILGHFYNWRTVALQATIPYIISMAIISTWPESPAWLISK : 192
BmStrGn1B : LLIYFGNNIPVLMIGRILGGMSAGGTVALGAIVIGEYSSPKYRGMYLNMKTASVCLGGMLVHILGHFYNWRTVALQATIPYIISMAIISTWPESPAWLISK : 192
BmStrGn2  : FNILLARNVSALLIARFNQGIAMGMSASLGPVLIGEYTSPNNRGAFLTSISLTIATGVLAVHTMGSYLSWQTTALVCAFIAFVDLLIVIYSPESPSWLADQ : 198
BmStrGn3  : LIFIAAKSFTALLVAKIFQGFSAGVESTTGYILVGEYVSPNHRASFLASFQPVMLFGDFITHSLSSICDWRNVAIILAFFSIPGLALIVFSPESPSFLAKI : 198
BmStrGn4  : IGFSLASSFPALLLAKIFQGMSIGLGTTMGGILIAEYSSPKYRGSLIATIPAALLVGVLSSHSLGLFYNWFQIAIILLFCSLPGLIIAVFTPESPTFLATK : 198
BmStrGn5  : ISFSLASSLSILLVAKIFQGMSIGLGTTMGGVLIAEYSSPKYRGSFTATIQTAMLSGSLIAHSFGLFYNWHQISTILVFFALLGLLIAVFSPESPSFLATK : 194
BmStrGn6  : ILFTLSSSFLELLIAKILQGITVGLGS-LGSNLIGEYSSPKYRASFIATIPTGLLFGELLIHCLGLICEWRDVALVIAFLCLPGLIASVFSPESPSFLVTK : 197
BmStrGn7  : ISITLSTGLTLLLIGRFLQGLSVGMGSCLIPIMIGEYTSSTRRGQFSMCMSVIMGLGTLAVHTVGSYYSWKITALVCSTITFFDLIIVILSPESPSWLAAQ : 198

BmStrGn1A : QQYEQSEKNFYFLRGKTEESYRELENMMQSQTNRVATKTKELTFT-------DKL----------------------------------------IIAEI : 245
BmStrGn1B : QQYEQSEKNFYFLRGKTEESYRELENMMQSQTNRVATKTKELTFT-------DKLVDFLKKFTKKNFLKPLFILLNGTILLETCGRHIFPAYALQIIAEI : 285
BmStrGn2  : GRYDECRKVFRWLRSEEEE--EELERMIEAAIVVRESKADVNLSESFLNKVKRNVSHFCSTIMKREFYRPIFIMMHIYTLGQWAGANILAAYTVDIFTAV : 296
BmStrGn3  : GMHDQCKKVFYWLRGPNEN--NEVEMLIKTNLPSKQNAINNDKIN-GYKKVIHKLSFIGTFFKKREVRISLFIMVHMQLINLFSGSILYDSYTVDIHTAV : 295
BmStrGn4  : GRYDECRKVFRWLRGSEDD--DELEKMIEADMIVKEAKKS-CKNN-LSKLIKQNLTYFVTSFKKREFYIPIFIVVHLNVINQFSGGIIHDSYPMDIHTAL : 294
BmStrGn5  : GRYDDCRKVFRWLRGPNEE--DELENMINTDMVVKETKKGRRKKT-TSKIIQKKLTYVITSFKKREFHIPVFIVIHLNAVNQFCGSLIHDIYAFDIHKAL : 291
BmStrGn6  : GRYDECRKVFRWLRGTDED--TELEAMIQTDKIIKETTKNYHKQS-LIKLFKAKLLYVLIVFRKREFRIPMIIMMHLNLINQFCGTFVNDMYTVDIHTAM : 294
BmStrGn7  : GRYDECRRVFRLLRTIDEE--EELDKMLAKSKELEDAK---IIRTGFMVKVKGGFSYINKTITRKEFYKPIFIMVHLFAMGQWAGINVLAPFTVNVIEII : 293

BmStrGn1A : TGS--KSQSFYYTMCIDVIITVSAVCSSILVKVMKRRTLLFSTGFAAFFVLIIVCAYLFLVAQGVIPDKYHWVPIALFWYFILANLGCTPIPLAFLGELFP : 345
BmStrGn1B : TGS--KSQSFYYTMCIDVIITVSAVCSSILVKVMKRRTLLFSTGFAAFFVLIIVCAYLFLVAQGVIPDKYHWVPIALFWYFILANLGCTPIPLAFLGELFP : 385
BmStrGn2  : IGE--DINIPLLIITLDAQRIISNAAAIFVIKKIKRRTMLFSTVTLNLFAFISTAAYTYCKGRGLLPFDHPVIGIALIHIHMFTIATGTVPLPFIIAGELFP : 396
BmStrGn3  : FGTDKDQYMYLIVMYLDIVRILSSIFSVFLTQKMKRRSMLLSLVGLNILMYLLLGIYVLCKNHNLLPFDHISIGIILYGFNYFSLAAGSVSLPNIVAGEIFP : 397
BmStrGn4  : YGT--DIYMFRVIASLDLQKIFATFLTIYLTSRIRRRPLYLTIVGLNILSLLCEAGYIYARRYNLLPFDHISIVIFLQHFHMFTSGAGGTSLTAIISNELYP : 394
BmStrGn5  : YGT--DIYMFKVIASLDIQRILSTVLTVYLTSRVRRRPLLFTLVGLNILAYICEAGYIYARRHSILPFDHMAIGVFLHHFHYFTNGAGSMSLPTIISCELFP : 391
BmStrGn6  : YGT--DAYMFQTAASLDVQRLVSTMLMIYVSSKVRRRPLLLALVGLNVLAYLLIAGYIYGRRNKILPFDHMAIGIILHHFHFFSIGTGSFPLVFIISGEIYP : 394
BmStrGn7  : VGK--DVNIPLITVAIDIQRIISSLFAVYIIKRIKRRTMLFSTLGLNAAVIFVTAAYVYFKSRGQLPFDHPMIGITLILIHMLSVATGSLPLPYTIAGEIFP : 393

BmStrGn1A : LEHRGAGSAVAGIFMSVILMLGLLLTPHLLAKIAVHGTFAVFGIIMGISLAILYVTLPETKDKTLQEIEDYFNYGR-RAYGPPDGELLPSPMDFSNPRS------ : 443
BmStrGn1B : LEHRGAGSAVAGIFMSVILMLGLLLTPHLLAKIAVHGTFAVFGIIMGISLAILYVTLPETKDKTLQEIEDYFNYGKFKDFKNVEEPDVKTKMLK----------- : 479
BmStrGn2  : LEFRSLAGGISVLFLSTNLFIAVKTFSLLLKSIGIHGAYLIYAGVVAYCLVIAGFFLPETKDRTLQEIEDEFRGRPLTVEELKSTQSLTSWKLRSQDRRCSSPVV : 501
BmStrGn3  : LANRSACGMICNITFSLYMFVNIKNVPYIFSGAGVSGVFFMNAALLSYALGMTMYTLPETKDKTLLEIEHILRG--CPIADDDETMINLKEHNETNF-------- : 492
BmStrGn4  : MQNRGLCGMICGAFFSIYMFLNIKSATYLFSLIGVDGTFCAYAALLFYSLVVFYYMVPETKDKTLQEIEDKLRG--YSIARRDETLKLKDAEL----------- : 485
BmStrGn5  : TADKGFCGLICSMVFSMFMFANIKSAPYLFALVGVEGTFCAYALLLLYCQVMLFYILPETKDKTLQEIEDKIRG--FSFADRVETLKTEEDELLS---------- : 484
BmStrGn6  : LTDKGYCVLFCSILYSIYMFTNIKTAPYLFSLIGIEGAFCVYAILLFYCLAVTLYLLPETKDKTLQEIENEFRG--YAIN--HETVKLNA--------------- : 480
BmStrGn7  : LEYRSLSSGISAVFCSINIFVSIKTLPYLFKTIGMHGAYCLYGVILVYCLTIAWFFLPETKDRTLQDIENEFRGKVTYPEDLKSAEPLTVWKS-NEEKAC----- : 491
```

Figure S8 Multiple amino acid sequence alignments of proteins encoded by *Gn_Str_*cluster members.

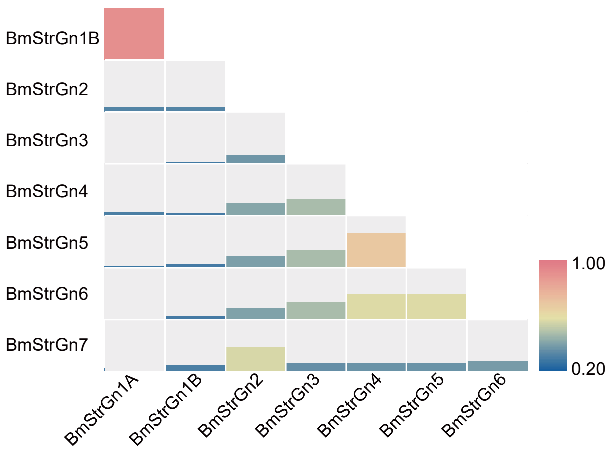

Figure S9 Amino acid sequence identity between Gn_Str_cluster members.

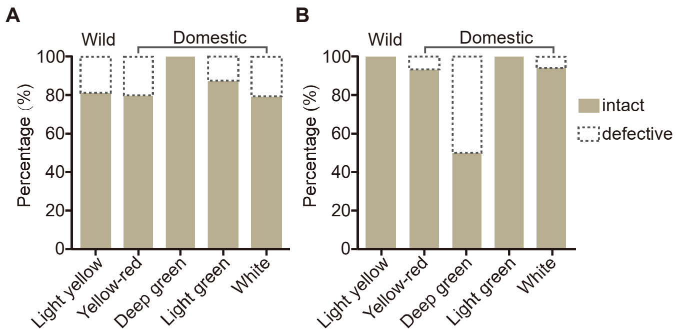

Figure S10 Genotype frequencies of *Gb* (A) and *Lg* (B) in each silkworm population.

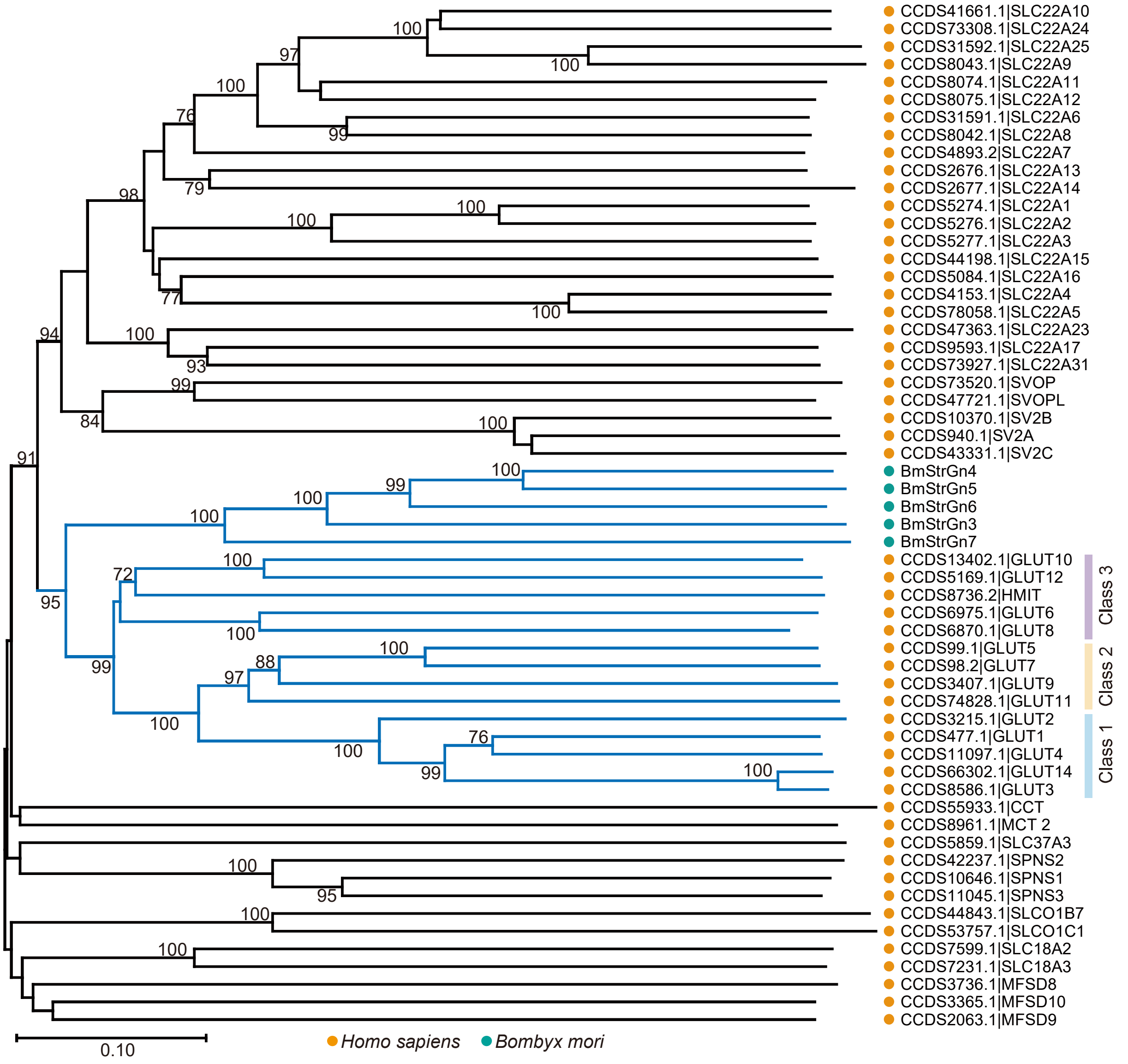

Figure S11 Phylogenetic tree of Gn_Strs and Strs encoded by the genome of *Homo sapiens*. The percentages of replicate trees in the bootstrap test (1000 replicates) are shown next to the branches. Only values greater than 70% are displayed.

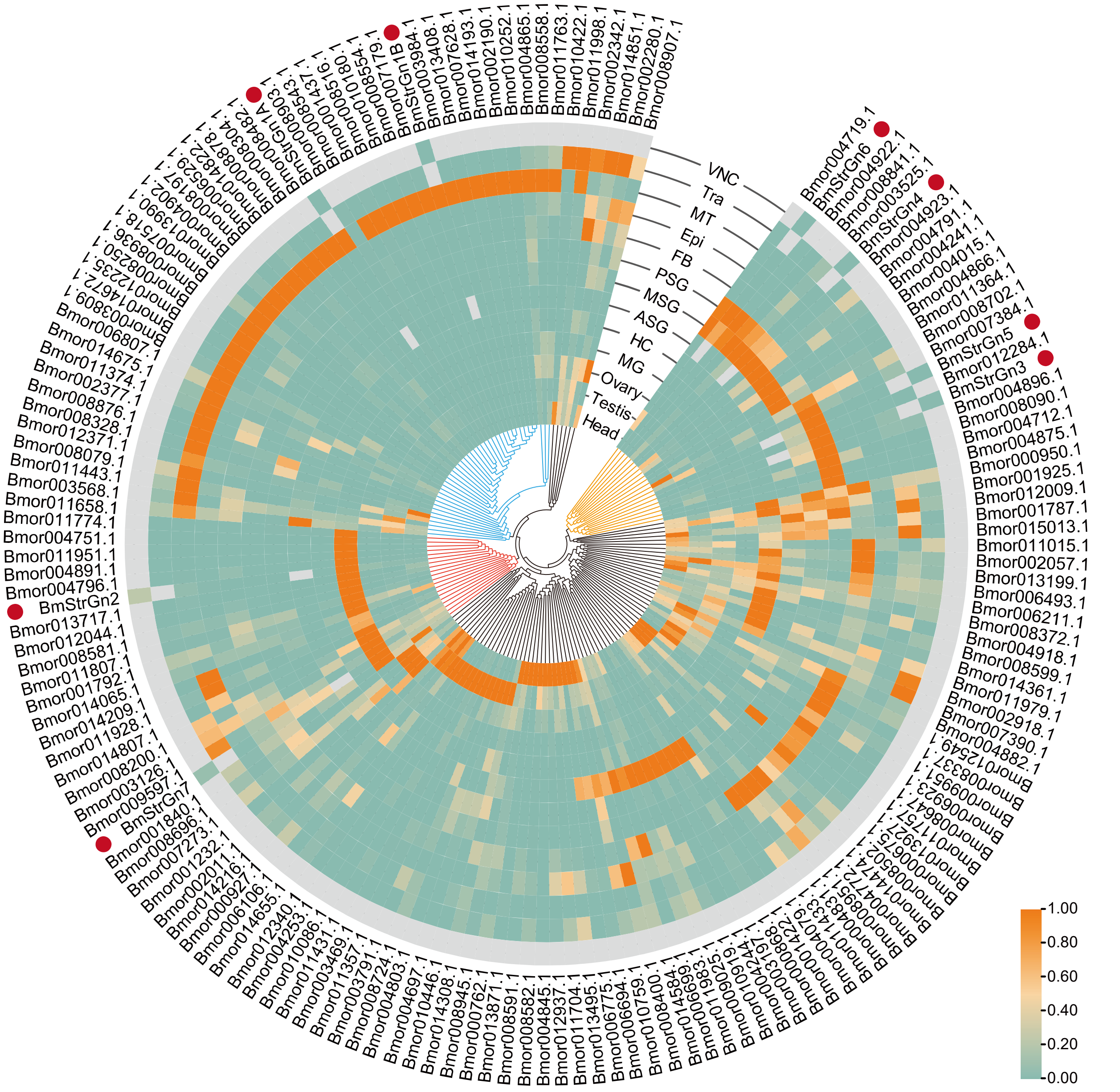

Figure S12 Spatial expression clustering of Strs encoded by the genome of *Bombyx mori*. Gn_Str_cluster members are marked with solid red circles. VNC, ventral nerve cord; Tra, Trachea; MT, Malpighian tubule; Epi, epidermis; FB, fat body; ASG, anterior silk gland.
